# Riboflavin photoactivation by upconversion nanoparticles for cancer treatment

**DOI:** 10.1038/srep35103

**Published:** 2016-10-12

**Authors:** E. V. Khaydukov, K. E. Mironova, V. A. Semchishen, A. N. Generalova, A. V. Nechaev, D. A. Khochenkov, E. V. Stepanova, O. I. Lebedev, A. V. Zvyagin, S. M. Deyev, V. Ya. Panchenko

**Affiliations:** 1 Federal Scientific Research Centre “Crystallography and Photonics” of Russian Academy of Sciences, Leninsky pr. 59, Moscow, 119333, Russia; 2 Shemyakin-Ovchinnikov Institute of Bioorganic Chemistry of the Russian Academy of Sciences, 16/10 Miklukho-Maklaya, Moscow 117997, Russia; 3 Institute of Fine Chemical Technologies, Moscow Technological University, 86 Prospect Vernadskogo, 119571 Moscow, Russia; 4 FSBSI “N.N. Blokhin Russian Cancer Research Center” of Ministry of Health of the Russian Federation, 24 Kashirskoye shosse, Moscow, 115478, Russia; 5 Laboratoire CRISMAT, UMR6508, CNRS-ENSIACEN, Universite Caen, 14050 Caen, France; 6 ARC Centre of Excellence for Nanoscale BioPhotonics, Macquarie University, North Ryde, NSW 2109, Australia

## Abstract

Riboflavin (Rf) is a vitamin and endogenous photosensitizer capable to generate reactive oxygen species (ROS) under UV-blue irradiation and kill cancer cells, which are characterized by the enhanced uptake of Rf. We confirmed its phototoxicity on human breast adenocarcinoma cells SK-BR-3 preincubated with 30-μM Rf and irradiated with ultraviolet light, and proved that such Rf concentrations (60 μM) are attainable *in vivo* in tumour site by systemic intravascular injection. In order to extend the Rf photosensitization depth in cancer tissue to 6 mm in depth, we purpose-designed core/shell upconversion nanoparticles (UCNPs, NaYF_4_:Yb^3+^:Tm^3+^/NaYF_4_) capable to convert 2% of the deeply-penetrating excitation at 975 nm to ultraviolet-blue power. This power was expended to photosensitise Rf and kill SK-BR-3 cells preincubated with UCNPs and Rf, where the UCNP-Rf energy transfer was photon-mediated with ~14% Förster process contribution. SK-BR-3 xenograft regression in mice was observed for 50 days, following the Rf-UCNPs peritumoural injection and near-infrared light photodynamic treatment of the lesions.

Riboflavin (Rf), also called Vitamin B2, is the main biochemical source of a flavin moiety in the cell, readily forming flavin mononucleotide (FMN) and flavinadenine dinucleotide (FAD), which play a vital role in the cellular metabolism. In pathological conditions, including oncogenesis, Rf metabolism and uptake are upregulated, sometimes at the expense of the Rf supply to the other tissues, as it was shown in the experiments with Rf-starved mice^1^. In humans bearing a breast cancer, the Rf carrier protein concentration in the cancer lesion was found to be markedly increased^2^. The accumulation of Rf in human breast cancer cells was shown to be specific, receptor-mediated^3^ and more efficient than that of folic acid broadly used as an endogenous cancer targeting moiety^4^.

The utility of flavin co-factor in enzymes is determined by its charge transport properties in the oxidation/reduction reactions. Flavins are photoreducible, i.e. capable of charge transfer per photon absorption, which mediates the cell signalling or gene expression in endogenous protein complexes, such as light-oxygen-voltage-sensing domains in bacteria and plants^5^. At the same time, biologically-unregulated photon-induced excitation of flavins in the ultraviolet-blue (UV-blue) spectral band can lead to formation of either singlet oxygen (^1^O_2_) via energy transfer to environmental oxygen (Type II, as shown in Fig. 1b), or hydrogen peroxide and derivatives via radicalisation (Type I)^6^ – altogether termed ROS and used hereafter. The ROS production property of Rf is known for a long time and used for antiviral and antibacterial disinfection^7^; for strengthening the corneal tissue in photorefractive surgery by the ROS-induced collagen cross-linking^8^. A fluorescent flavoprotein miniSOG, with FMN as the cofactor, was shown to generate ^1^O_2_^9^, whereas its recombinant analogue fused with a targeting mini-antibody 4D5scFv appeared to be a potent immune-photosensitiser for photodynamic therapy (PDT) of human breast adenocarcinoma cells^10^. Rf was also shown to suppress the solid tumour growth by inhibition of the expression of the tumour factors, which was explained by the photodegradation-induced phototoxicity^11^.

The FDA-approved riboflavin non-toxicity, its goodness as a dietary supplement (Vitamin B2) combined with the phototoxicity and cancer tropism would make it a PDT drug of choice, provided its poor photoactivation depth due to the high absorption of biological tissue in the UV-blue range is ameliorated. Emerging nanomaterial termed upconversion nanoparticles (UCNPs)^12^ provides a solution to this problem. UCNPs are capable to convert deeply-penetrating near-infrared (NIR) light at a wavelength of 975 nm to UV-visible light to excite photosensitive PDT drugs^13^,^14^. The UCNPs conversion efficiency (*η*_*UC*_) in the UV-blue spectral range, however, remains notoriously low, despite the most recent advances in this direction^15^. We report on the rational design of UCNPs to yield unprecedentedly high *η*_*UC*_ in the UV-blue range (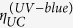) that enabled demonstration of the NIR-induced phototoxicity of Rf in cells and human breast cancer xenografts.

## Results

### Rf accumulation and photocytotoxic action

We characterised the photophysical and phototoxic properties of Rf using cell cultures and live laboratory animals, aiming to demonstrate its potential for clinical postoperative treatment of cancer lesions.

**Figure 1:**
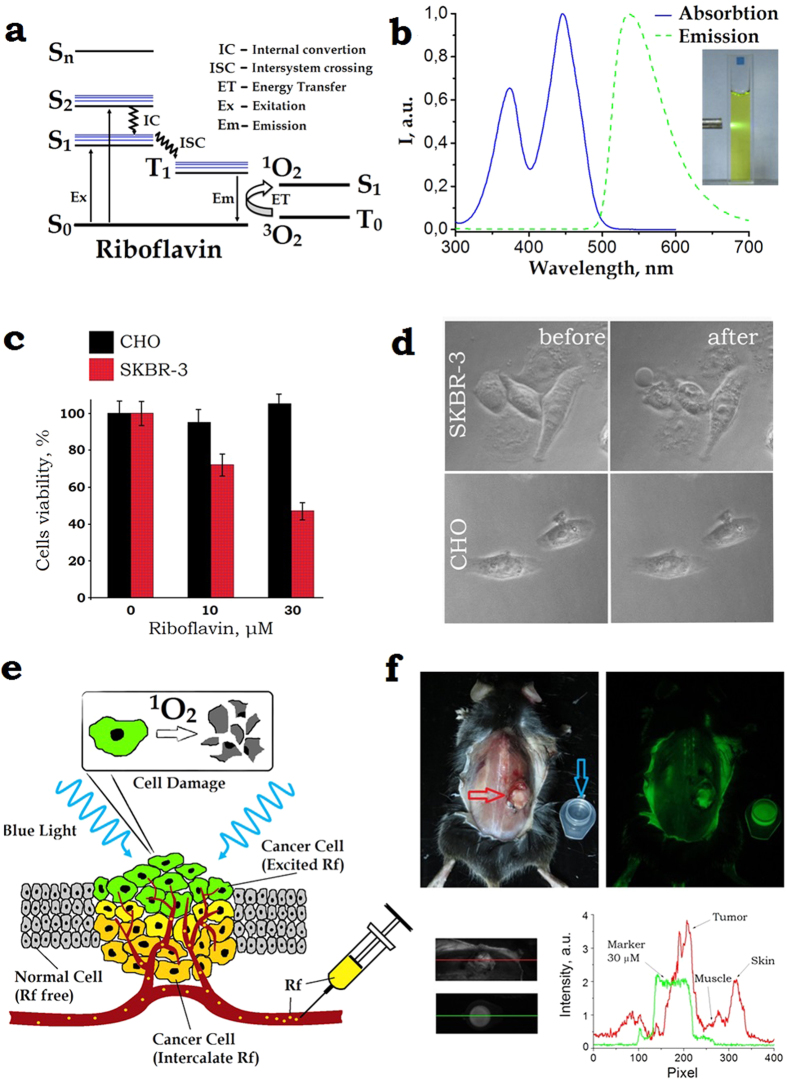
(**a**) Schematic diagram of the energy levels of riboflavin. A photon excites Rf from the singlet ground state (S_0_) to the first excited singlet state (S_1_) from which it can either decay to the S_0_ or undergo intersystem crossing to an excited triplet state (T_1_). Rf in the long-lived T_1_ state is capable to non-radiatively (collisionally) drive environmental oxygen from its triplet ground state ^3^O_2_ to the chemically reactive singlet excited state ^1^O_2_. The excitation and emission spectra of Rf are shown in Panel (**b**). (**c**) The MTT assay of the cells after incubation with Rf for 90 min followed by 365-nm light exposure at the dose 4.2 J/cm^2^. SK-BR-3 and CHO cells are marked red and black, respectively. The riboflavin *C*_*Rf*_ ≈ 30 μM reduced the SKBR-3 cells viability to 47 ± 7%. Error bars represent standard deviation (SD) for three independent experiments. (**d**) Phase-contrast images of SK-BR-3 and CHO cells treated with 30-μM riboflavin, before (left) and 2-h after (right) 10-min 365-nm light irradiation. Rapture of SK-BR-3 cells membrane was evident (bubbles), whereas the control CHO cells showed no morphological changes. (**e**) Schematic representation of the Rf-aided photodynamic treatment of superficial layers of targeted cancer cells shown as polymorphic yellow/green-coloured cells embedded into a layer of monomorphic grey-coloured normal cells. Buffer solution of Rf is injected into the tumour interstitium. ^1^O_2_ generated in the superficial cancer cell layer (green-coloured) exposed to UV-blue light induce phototoxicity. (**f**) **Top row:** left and right panels, *post mortem* bright-field and epi-luminescent images of BDF1 mice with a Lewis lung carcinoma tumour grafted on the dorsal side (marked by a red arrow), respectively. A plastic cuvette filled with *C*_*Rf*_ ≈ 30 μM is shown besides the animal (marked by a blue arrow). The epi-luminescent image was acquired under 450-nm excitation; fluorescence detection spectral band was 500–570 nm. **Bottom row:** bright-field zoomed images of the grafted tumour and cuvette and its fluorescence signal intensity profiles.

Riboflavin (Sigma-Aldrich^©^) and its highly water-soluble form, flavin mononucleotide (Pharmstandart^©^) used in our experiments are characterised by a broad UV-blue absorption band with two peaks at 375 and 450 nm, corresponding to the singlet S_2_ and S_1_ excited states (see Fig. 1a), respectively, which gives FMN its characteristic yellow-orange colour (Fig. 1b, inset). The excited states can decay to a long-lived triplet state T_1_ via an intersystem-crossing transition from where non-radiative (collision) energy transfer to an environmental oxygen molecule is likely to occur, driving it to the singlet state (^1^O_2_) (details are provided in Supplementary Information, SI 1). In aerated solution, FMN generates more singlet oxygen (quantum yield, 0.51 ± 0.07) than exogenous photosensitisers, such as clinically used Photofrin^16^.

The photocytotoxic action of Rf occurs predominantly via photogeneration of ^1^O_2_^16^. Figure 1c shows the results of the colorimetric MTT assay (see M&M for details) for assessing the viability of targeted SK-BR-3 and control CHO cells, following the cell culture incubation with Rf, washing cycles and cells exposure to 365-nm light from a light-emitting diode with the intensity of 7 mW/cm^2^ for 10 min. At the concentration of Rf *C*_*Rf*_ ≈ 30 μM in the culture medium, the SK-BR-3 cell viability dropped to 47 ± 7% of the level recorded for cells maintained in dark. We hypothesise that the 30-μM concentration of Rf in the cell incubation medium (equivalent to the extracellular environment in tumour interstitium) sufficed to cause breast adenocarcinoma cell death, sparing the surrounding normal tissue modelled by the control cells CHO. This was partly explained by the high uptake of Rf by SK-BR-3 cells, which was measured to be 3- and 1.5-fold greater than that of the control CHO cells and fibroblasts, respectively (see SI 2). The microscopy observations of the cells 2 h after the light exposure revealed disruption of the SK-BR-3 cell membranes visualised as bubbles bulging out of each damaged cell (Fig. 1d). In order to pinpoint the PDT-induced death mechanism of cells pre-incubated with Rf, we carried out a apoptosis assay by flow cytometry for 6 h, 12 h, 24 h time points and immunofluorescence assay of the Caspase-3 activity. The PDT-treated cells pre-incubated with Rf displayed an increased level of active Caspase-3, which was comparable with that of the positive-control cells treated with etoposide. At the same time, the level of active Caspase-3 in the negative-control sample (untreated cells) was ~2-times lower. Taken together, these results indicated that apoptosis was the most likely mechanism of cell death pathway in the PDT-treated group of cells pre-incubated with FMN (see SI 3 for details). At the same time, CHO cells remained morphologically intact and viable.

In order to demonstrate that the high phototoxic value of *C*_*Rf*_ is attainable in tumour interstitium, we carried out epi-luminescent imaging of a laboratory mouse bearing an induced Lewis lung cancer (see M&M). 3 mg of FMN (10 mg/mL, PBS buffer) was injected intravenously and 12 hours later the euthanised animal with a cancer site exposed surgically was imaged using a home-built epi-luminescent imaging system^17^ (detailed in SI 4). A cuvette filled with 30-μM FMN aqueous solution was placed nearby for comparison (see Fig. 1f). A fluorescence signal originated from the cancer lesion (shown by an arrow) exceeded that of the Rf reference cuvette by almost two-fold, implicating that the threshold PDT concentration of Rf was attainable in living organisms. Comparison between the FMN fluorescence signals in biological tissue and clear solution of known concentration was carried out to establish a correspondence between the FMN concentration and its detected fluorescent signal. The calibrated FMN concentration was underestimated due to the Rf binding to biomolecular species in cells causing the reduction of the fluorescence quantum yield of Rf. In addition, the biological tissue absorption and scattering in blue spectral range caused attenuation of both excitation and emission light, although these effects were minimised due to the measurements were confined to a superficial subcutaneous layer with the skin flap removed. The Rf concentration achieved in the tumour interstitium was at least 60 μM, with the contrast of ~5 with respect to the subcutaneous tissue background. Such high contrast is acceptable for PDT to treat the cancer lesion selectively minimising phototoxicity impact to the surrounding normal tissue^18^.

### Rf photoactivation by UCNP

In order to translate the riboflavin-mediated PDT from cancer cells to cancerous tissue, one needs to deliver UV-blue excitation light to the full volume of an entire cancer lesion. This is impeded by the high extinction of biological tissue in this spectral range. For example, 340-nm light penetration depth was estimated as 60 μm, reaching the topmost layer of the viable epidermis layer in human skin^19^. The limited treatment range has been recognised as a problem, driving PDT drug design towards the longer (NIR) excitation wavelengths^20^, where the biological tissue extinction is minimal. For the Rf photosensitisation, one needs to devise means of conversion NIR to UV-blue light at the sub-centimeter depth. Upconversion nanoparticles provide these means. Despite a number of reports on the photoactivation of fluorophores by UCNPs^14^,^21^,^22^, the present challenge of the photoactivation of Rf in the UV-blue spectral band is unmet by the state-of-the-art UCNPs^15^, and demands a purpose-design of UCNP to kindle its UV-blue photoluminescence.

**Figure 2:**
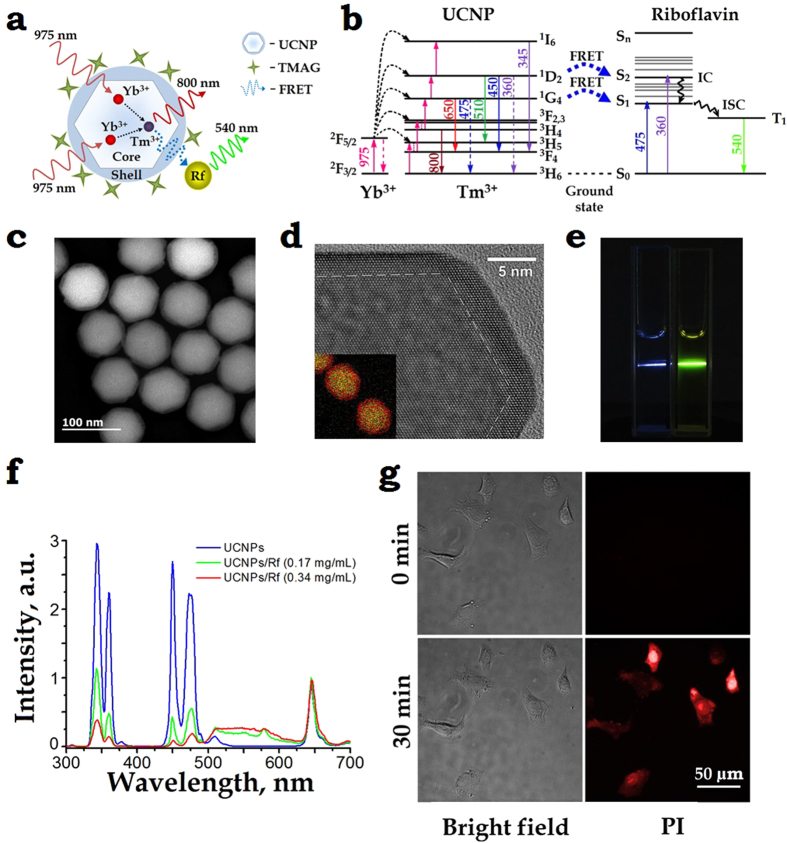
(**a**) A schematic diagram of the core/shell UCNP, explaining the 975-nm excitation (solid red wavy lines) of Yb^3+^ ions in the core, which then non-radiatively transfers (dashed arrows) the energy to an Tm^3+^ ion that passes the energy (dashed blue wavy line) to an Rf molecule via a resonant energy transfer (RET) process. The UCNP surface is coordinated by tetramethylammonium hydroxide (TMAH). (**b**) Energy level diagram of a UCNP – Rf pair. Excitation at 975 nm drives Yb^3+^ to the excited state ^2^F_5/2_ from which it can non-radiatively transfer the energy to Tm^3+^ (via ^3^H_6_ → ^3^H_5_ transition, followed by the relaxation to the metastable level ^3^F_4_). Two metastable excited state ^2^F_5/2_ Yb^3+^ and ^3^F_4_ Tm^3+^ ions coalesce to drive Tm^3+^ to the ^3^F_2,3_ state, while an Yb^3+^ decays to the ground state ^2^F_3/2_. Likewise, collective energy process of ^2^F_5/2_ Yb^3+^ and ^3^F_4_. ^3^H_4_. ^1^G_4_ … Tm^3+^ drives Tm^3+^ to the ^3^H_4_.^1^G_4_. ^1^D_2_ …, respectively (magenta arrows). There is a probability to populate S_1_, S_2_ levels of Rf via RET or Föster RET processes from the ^1^G_4_, ^1^D_2_ levels of Tm^3+^. (**c**) High-angle annular dark-field scanning and (**d**) high-resolution TEM images of as-synthesised core/shell NaYF_4_:Yb:Tm/NaYF_4_ UCNPs mean-sized 75 ± 5 nm, featuring the β-crystal phase. Overlay Y (green) and Yb (red) elemental EDX mapping of UCNPs is given as a bottom left insert in (**d**). (**e**) *In vitro* demonstration of RET of a UCNP–Rf donor-acceptor pair. Two cuvettes filled with plain UCNPs and UCNP-FMN 0.34 mg/mL aqueous colloids illuminated with a 975-nm laser beam. The respective blue and yellow traces of photoluminescence illustrate a strong RET effect. (**f**) Spectra of UCNP- FMN in water under 975-nm excitation acquired at 0, 0.17 mg/mL and 0.34 mg/mL concentrations of Rf (blue, green, red curves), respectively, with the concentration of UCNPs 0.5 mg/mL. A broadband fluorescence signal from 500 nm to 620 nm ascribed to the FMN emission was a strong manifestation of the RET between UCNP and FMN. (**g**) Demonstration of the phototoxicity effect of UCNP-Rf pair on SK-BR-3 cells irradiated with a 975-nm laser. Phase contrast (left) and propidium iodide (PI) fluorescence (right) images of the cells before (top images) as compared to the cells after (bottom image) irradiation highlights disintegration of the cells membranes.

UCNP represents an inorganic nanocrystal matrix of the most popular composition NaYF_4_ co-doped with ytterbium (Yb^3+^) and thulium (Tm^3+^) lanthanide ions. In the NaYF_4_:Yb:Tm quantum system, a network of closely spaced Yb-ions sensitises NIR radiation at 970–980 nm and resonantly and non-radiatively couples this energy to neighboring Tm-ions. A Tm-ion has multiple excited states with exceptionally long (sub-ms) lifetimes (see Fig. 2a). One excited Tm-ion can be transferred to the next excited energy level at the expense of the other participating adjacent Yb-ion decaying to the ground state via an energy transfer upconversion process. Eventually, Tm^3+^ radiates in 800-nm (two sequential photons), 475-nm (three sequential photons), 360-nm and 450-nm (four sequential photons) and 345-nm (five sequential photons) spectral bands (Fig. 2a). Obviously, the UCNP excitation/emission process is nonlinear, whereby its conversion efficiency^23^ *η*_*up*_ grows versus the excitation intensity reaching saturation at the high excitation intensity ranging from 10 to 100 W/cm^24^. This value is nevertheless ten-thousand-fold smaller compared to that used in the widespread nonlinear optical microscopy making the choice of UCNP as the photon energy upconversion agent very attractive. In many cases, the photoexcitation process favours the population of the lower energy levels of UCNP, i.e. ^3^H_4_ of Tm^3+^, due to the effects of crystal matrix phonons, environmental molecules (e.g. OH moieties)^25^, or high excitation intensities^26^, which non-radiatively depopulate the higher-energy levels.

We addressed the problem of the reportedly low conversion efficiency of UCNP in the UV-blue band, 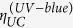 by synthesising UCNP of the core/shell architecture using a thermal decomposition method^27^, where the key parameters were the optimised dopant composition and reaction kinetics (see M&M for details). The UCNP core represented a fluoride of the hexagonal crystal phase, β-NaYF_4_ codoped with the Yb^3+^ and Tm^3+^ ions in 18% and 0.6% molar ratios, respectively; and undoped crystal shell NaYF_4_ (see TEM images, Fig. 2c,d), in a short-hand notation β-NaYF_4_:Yb:Tm/NaYF_4_. As a result, an unprecedentedly high value of 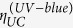 = 2.0 ± 0.2% at the excitation intensity of 50 W/cm^2^ was achieved, while the integral *η*_*UC*_ reached 9.5 ± 0.2% (detailed in SI 5).

Two UV-blue photoluminescence doublet bands (see Fig. 2f, blue curve) fall squarely into the Rf (or FMN) absorption band, thus enabling design of an energy transfer donor-acceptor pair UCNP-Rf, relying on resonance energy transfer processes (RET). Among these, light resonance energy transfer (LRET) relies on the energy transfer from the donor to the acceptor via photons, hence the process is long-range. In the Förster RET process (FRET), the donor transfers energy non-radiatively and efficiently (measured in tens of %) provided the acceptor, located at the nanometre proximity, with the process probability decaying as *r*^−6^ (*r*. donor-acceptor distance).

Placement of FMN molecules within the range of <10 nm to emitting ions (Tm^3+^) in UCNP presents a challenge (as discussed in SI 6), since it compromises the need to shield UCNP from emission-quenching environment, and is furnished by the core/shell UCNP architecture, as reported here. The shell thickness of 3–10 nm separates the donor-acceptor Tm^3+^-FMN pairs^15^, this is aggravated by an additional coating with amphiphilic polymer^28^ or silica shell^29^. Such configuration of β-NaYF_4_:Yb:Tm/NaYF_4_ coated with a layer of amphiphilic polymer, poly(maleic anhydride-*alt*-1-octadecene) (PMAO), was attempted in this work, and exhibited a low-yield RET (as detailed in SI 7). A donor architecture tailored for the more efficient FRET was based on tetramethylammonium hydroxide (TMAH·5 H_2_O, molecular weight 181) surface-modified UCNPs^17^. The partial displacement of hydrophobic oleic acid moieties from the surface of as-synthesised UCNPs with low-molecular-weight TMAH (see M&M) stabilised them in aqueous and physiological solutions, preserved 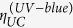 and made UCNP surface accessible for small molecules of Rf.

We tested the FRET effect of UCNP–FMN donor-acceptor pair by incubating TMAH surface-modified UCNPs and FMN in a water-filled cuvette under 975-nm excitation (see Fig. 2e). UCNP-FMN spectra were acquired at the FMN concentrations of 0, 0.17 and 0.34 mg/mL (blue, green, red curves), respectively, while the concentration of UCNP was maintained at 0.5 mg/mL (see Fig. 2f). Under these conditions, several molecules of FMN occur in the FRET-vicinity of a UCNP particle at any instant of time, so that acceptors changed for each donor. A broadband fluorescence signal from 500 nm to 650 nm ascribed to the FMN emission manifested the energy transfer between UCNP and FMN. The RET-mediated energy depletion of UCNP was profound in the UV-blue band, especially at 360 nm and 475 nm, corresponding to the ^1^G_4_ → ^3^H_6_ and ^1^D_2_ → ^3^H_6_ transitions. In order to quantify a relative contribution of the FRET to overall RET, we measured a decrease of the UCNP emission lifetime (*τ*_*UP*_) for the main UV-blue bands in the presence of Rf acceptor molecules. *τ*_*UP*_ of ^1^G_4_ → ^3^H_6_ (475 nm) and ^1^D_2_ → ^3^H_6_ (360 nm) in the presence of acceptors was changed from 645 ± 5 μs to 575 ± 5 μs and from 317 ± 4 μs to 310 ± 4 μs, respectively. Firstly, this measurement proved that FRET process did take place; secondly, the corresponding FRET efficiencies were evaluated as 11% and 3%, totalling 14% (for details see SI 6).

The phototoxic action of UCNP-FMN pair on SK-BR-3 cells *in vitro* was demonstrated by a two-phase incubation procedure: Firstly, TMAH-UCNPs were immobilised on the cell plasma membrane at room temperature, immediately followed by incubation with 100-μM FMN and irradiation with 975-nm laser light, aiming to achieve an irradiation dose of 600 J/cm^2^ (for details see SI 8 and 9). This resulted in the generation of ROS eventually inducing cell death, which was probed by propidium iodide (PI), with the result shown in Fig. 2g. It is clear that 30 min after irradiation cells become permeable to PI, which is observable in the red fluorescent channel and indicates that cells death occurred as a result of ROS-mediated membrane disintegration.

### NIR PDT treatment

**Figure 3:**
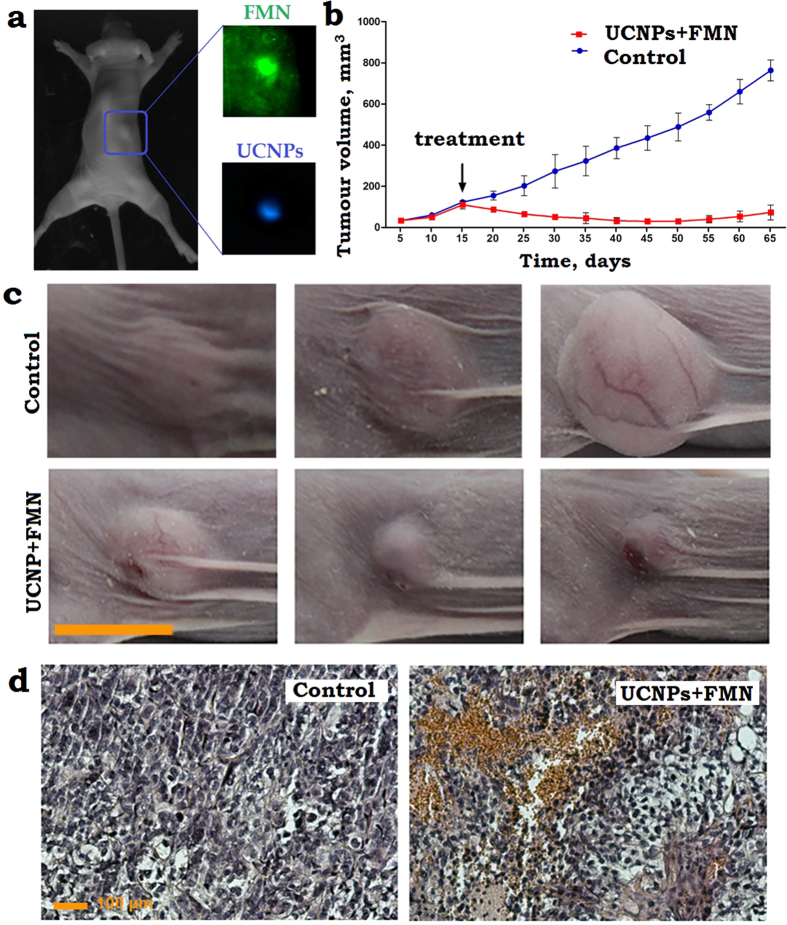
(**a**) A photograph of the dorsal side of the immunodeficient mouse, bearing a grafted subcutaneously SK-BR-3 tumour 15 days post-implantation. A PBS solution of FMN and UCNPs was injected peritumourally. The tumour area is marked by a (blue) rectangle, its zoomed-in images spectrally filtered to emphasise FMN and UCNPs emissions are shown in insets labelled FMN and UCNPs, respectively. In FMN and UCNP images, the tumour exhibited contrast of 2 and 30, respectively, demonstrating the superiority of UCNP-assisted imaging. (**b**) A plot of the SK-BR-3 tumour evolution, showing progressive stable growth of the control tumours (non-irradiated) and tumour regression post-PDT treatment (black arrow, day 15) using FMN + UCNPs. (**c**) A time-lapse series of the bright-field photographs of the SK-BR-3 tumour area taken prior to the 975-nm laser treatment “15”, 25 and 50 days after treatment and the photographs of appropriate controls. Scale bar, 10 mm. (**d**) Histological images of the tumour tissue sections stained with hematoxylin and eosin, excised 1 day after the 975-nm PDT treatment. FMN + UCNPs in PBS solution were injected peritumourally in the control and PDT treated tumours. FMN + UCNPs after irradiation display profound hemorrhages, respectively, whereas control shows no abnormalities. Scale bar, 100 μm.

We carried out a series of experiments on SKBR-3 xenograft bearing mice aiming to demonstrate the feasibility of UCNP-Rf photosensitised PDT treatment. Approximately, 2 × 10^6^ SK-BR-3 cells were inoculated subcutaneously into ten immunodeficient Balb/c nu/nu mice (see M&M for details) and grown for 15 days until tumours reached 120 ± 15 mm^3^ in volume, as shown in Fig. 3a. 50 μL of PBS solution containing 500 μM FMN (Pharmstandart^®^) and 25 μg UCNPs (termed FMN + UCNP) was injected into the tumour-surrounding tissues and incubated for 1.5 h, followed by irradiation with a 975-nm of the dose 900 J/cm^2^ (average intensity was limited to 1.5 W/cm^2^ to prevent tissue overheating). Laboratory animals, which were administered FMN + UCNP peritumourally, without subsequent laser exposure, were used as the control. The *in vivo* NIR-induced PDT efficacy was assessed by measuring the tumour volumes over a period of 50 days, with the results presented in Fig. 3b,c. A plot of the SK-BR-3 tumour evolutions shows a progressive growth of the control untreated tumour from the day 20, following a typical dormancy during the incubation period, whereas obvious tumour volume regressions were observed in case of the PDT treatment using FMN + UCNP photosensitiser. The tumour growth inhibition was estimated as 90 ± 5% of volume on the day 50 after PDT treatment.

After 24 hours of the PDT treatment, three mice were sacrificed for histological analysis (Fig. 3d). The control group was characterised by the presence of healthy cells without any pathological changes. However, the FMN + UCNP-injected tumours with subsequent 975-nm radiation treatment displayed a dramatic decrease in the density of nucleated cells. In addition, a fraction of the tumour area was filled with eosinophilic material or empty spaces devoid of any materials. In addition, the PDT treatment caused the rupturing of blood vessels with extensive hemorrhages into the tumour interstitium. Therefore, the peritumoural administration of FMN + UCNP, followed by the 975-nm laser exposure exhibited considerable tumouricidal effects in the investigated SK-BR-3 xenografts amplified by pathomorphological modifications of the cancer lesion matrix. In order to confirm that the successful PDT effect was due to the coalescence of the main PDT components, UCNPs, FMN and 975-nm laser irradiation, we performed a number of control experiments (see SI 10 and 11), with the statistical results presented in Figure S11. Based on this histological analysis, we estimated the range of the UCNP-FMN pair-assisted PDT treatment depth as 4–6 mm considering the thickness of the tumour lesion (see SI 12).

## Conclusion

In conclusion, we have demonstrated that dietary supplement Riboflavin (Vitamin B2) can be photosensitised selectively and efficiently to kill human breast adenocarcinoma cells SK-BR-3 and exert tumouricidal effect on grafted SK-BR-3 tumours in immunodeficient mice. In order to extend the treatment depth ~10-fold in biological tissue to 4–6 mm, we introduced rationally-designed upconversion nanoparticles, with the conversion efficiency in the UV-blue spectral range of 
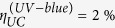
, and minimal hydrophilic surface dressing that were capable to efficiently convert deeply-penetrating near-infrared light into UV-blue light by resonant energy transfer processes to photosensitise Rf using the moderate intensity and doses of NIR light acceptable for PDT procedures. The introduced biofunctional hybrid nanomaterials hold promise for a novel photoactivatable theranostics platform. In the broader prospects, this work introduces a new approach to trigger photo-biological processes at the centimetre-range depth in live biological tissue, including cross-linking, optogenetic transducers and other light-control processes.

## Materials and Methods

### Cell culture

Cell lines SK-BR-3 (human breast adenocarcinoma) (HTB-30™; ATCC) and CHO (Chinese hamster ovary) cell lines were grown as a monolayer at 37 °C in a 5% CO2 atmosphere and 100% humidity. RPMI growth medium was supplemented with 10% fetal bovine serum (HyClone) and 2 mМ L-glutamine (PanEko).

### MTT assay of the cell viability

The MTT assay involves the conversion of MTT (3-(4,5-dimethylthiazol-2-yl)-2,5-diphenyltetrazoliumbromide) in mitochondria by succinic dehydrogenase into an insoluble violet formazan, which is then solubilised in DMSO. Briefly, cells (5 × 10^4^ per ml) were seeded in 96-well plates in 0.15 ml of culture medium, and 24 h later were treated with riboflavin in different concentrations in the dark. The medium was removed 90 minutes later and the cells were washed with PBS twice. The cells in wells containing 0.15 ml of PBS were irradiated at 365 nm for 10 min with the power density of 7 mW/cm^2^. Then, PBS was removed and the medium was added. After 72-h incubation, 100 μl of MTT (with a final concentration of 0.5 mg/ml) was added. The formazan crystals were dissolved in DMSO, and the absorbance was measured with a microplate reader (Awareness Technology Inc., USA) at the 540 nm wavelength.

### Cell imaging post incubation with Rf

SK-BR-3 and CHO cells were grown in a 96-well plate overnight and incubated in a culture medium supplemented with 30 μМ Rf. After 90 min, the cells were washed with PBS buffer and exposed to optical radiation at 365 nm of the dose 4.2 J/cm^2^. The phase-contrast images were taken 2 h post irradiation with an inverted fluorescent microscope Axiovert 200 (Zeiss, Germany).

### Synthesis of β-NaYF4:Yb3 + Tm3 + /NaYF4 UCNPs

The synthesis of lanthanide doped NaYF_4_ UCNPs is based on the coordinate stabilization of yttrium, ytterbium, and thulium metal salts in a solution of oleic acid and octadecene carried out with heating in an oxygen-free atmosphere. The mixture of Y_2_O_3_ (0.794 mmol), Yb_2_O_3_ (0.2 mmol), Tm_2_O_3_ (0.06 mmol) was suspended in 10 ml 70% trifluoroacetic acid and gently refluxed until a clear solution was obtained (≈6 h), then cooled to room temperature. Solution was evaporated and residue was dried in vacuum (0.1 Torr) for 1 h. This rare-earth trifluoroacetate mixture and sodium trifluoroacetate (2 mmol) were added to 10 ml oleic acid and 10 ml 1-octadecene in three-neck flask equipped with the thermometer and glass magnetic stirrer. The solution was heated up to 120 ^o^С and stirred under vacuum for 30 min for degassing and water removing. Then, the mixture was gradually heated at a rate of 200 ^o^С/min to 320 ^o^С on Wood’s alloy bath and incubated at this temperature for 30 min in argon atmosphere. Then, the mixture was cooled by adding 15 ml 1-octadecene. In order to cool to room temperature 150 ml propanol-2 was added and mixture was centrifuged at 6000 rpm for 20 min. Obtained nanocrystals were purified by absolute ethanol addition, dried, and dissolved in 10 ml oleic acid and 10 ml 1-octadecene. Yttrium trifluoroacetate (previously prepared from 0.5 mmol Y_2_O_3_ and 5 ml 70% CF_3_COOH, as described above) and sodium trifluoroacetate (1 mmol) were added, mixture was heated up to 120 ^o^С and stirred under vacuum for 30 min. Then, the mixture was heated up to 305 ^o^С, incubated for 15 min on Wood’s alloy bath in argon atmosphere and cooled to room temperature. Obtained core/shell nanoparticles were centrifuged at 6000 rpm for 20 min. The product of synthesis is hydrophobic monodisperse nanoparticles (75 ± 5 nm) with a core/shell structure (β-NaYF_4_:Yb^3+^Tm^3+^/NaYF_4_) capable of forming stable colloids in non-polar organic solvents such as hexane and chloroform.

### Transmission Electron Microscopy (TEM)

High angle annular dark field (HAADF) scanning TEM (STEM) and high-resolution TEM studies were performed using a JEOL ARM200F cold FEG double aberration corrected electron microscope operated at 200 kV and equipped with a large solid-angle CENTURIO EDX detector and Quantum EELS spectrometer. TEM sample was prepared by dissolving sample dissolution in methanol and deposition on hollow carbon Cu grid.

### TMAH-modified UCNPs

UCNPs were modified with tetramethylammonium hydroxide (TMAH) in order to obtain aqueous dispersions of UCNPs. We employed the ligand exchange reaction as we reported in ref. 17. In general, ligand exchange reaction was carried out with TMAH, which is a low molecular phase transition catalyst. TMAH is dissociated in water by producing OH^-^ ions, which are absorbed on the surface of UCNPs, partly displacing oleic acid moieties from the UCNP surface. The UCNP modification procedure consisted in preparation of UCNP dispersion in aqueous solution of TMAH followed by solvent evaporation.

### Visualization system

The custom-developed epi-luminescent optical imaging system was used to visualize the accumulation of Rf and UCNPs at the tumour site. The test object was scanned by the cw semiconductor laser at 450 nm or 975 nm via 2-axis laser beam deflection unit Miniscan-07 (Raylase, Germany). The detection of the photoluminescent signal was performed using Falcon EMCCD camera (Raptor Photonics, Northern Ireland), equipped with the F = 0.95 objective. The interference filters (Semrock, USA) were placed in front of the lens, cutting the exciting laser radiation. Detailed description of used setup can be found in SI 4.

### Demonstration of the phototoxicity effect of UCNP-riboflavin pair

SK-BR-3 cells were seeded in a 96-well plate at the density of 5 × 10^4^ cells per ml of cell culture medium and incubated overnight. Then the cells were treated with UCNPs at the concentration 1 μg/ml for 10 min and washed with PBS. Then the solution of 100 μM FMN and 2 μM propidium iodide dissolved in PBS were added to the cells in the dark and the first snap of the cells was taken (zero point). A green filter was used in order to avoid riboflavin photo activation. Then the cells were irradiated with 975-nm laser of the dose 600 J/cm^2^. The second snap was taken after irradiation.

### Animal experiments

The experimental animals were housed under controlled environmental conditions (constant temperature, humidity, and a 12 h dark–light cycle) and allowed access to water and mouse chow freely. All animal experiments were performed in accordance with European and Russian national guidelines for animal experimentation and animal experiments were approved by the local animal and ethics review committee of the FSBSI “N.N. Blokhin Russian Cancer Research Center”.

### Lewis lung cancer mouse model and Rf injection procedure

Lewis lung cancer (LLC) of a laboratory mouse model BDF1 (C57Bl/6 × DBA2) was used to assess the Rf (FMN) accumulation in cancer lesions *in vivo*. LLC was extracted from BDF1 mice on the day 11 after grafting. The extracted tumour was shredded with scissors and suspended in DMEM in a 1:10 ratio. This DMEM suspension of LLC of 0.3 mL in volume was grafted subcutaneously to BDF1 mice with an average weight of 30 mg. In ten days a buffer solution of FMN were injected into the mice intravenously (through a retro-orbital sinus). It was evaluated that 3 mg of FMN was required to achieve the interstitial FMN concentration of 60 μM, as shown in Fig. 1f.

### SK-BR-3 xenograft mouse model

SK-BR-3 cells (ATCC HTB-30) were cultured in RPMI-1640 medium containing 10% FBS, 2 mM L-glutamine, 100 U/ml penicillin, and 100 μg/ml streptomycin. Cells were treated with trypsin when near confluence and harvested. Cells were pelleted by centrifugation at 1,200 rpm for 5 min and resuspended in sterile DMEM. SK-BR-3 cells (2.0 × 10^6^ cells in 200 μL DMEM) were implanted subcutaneously into the right flank of the mice. Tumour volume was estimated by the standard method of calipation and was calculated by the following formula: V = (length × [width]^2^)/2, assuming a hemi-ellipsoid shape. Antitumour activity of UCNPs and riboflavin was determined by evaluating the tumour growth inhibition rate (TGI%) calculated as: [TGI%] = (TG_control_ − TG_test_)/TG_control_ × 100%.

### Histological sample preparation procedure

Samples of xenograft tumours were fixed in 4% buffered formaldehyde for 24 h, processed into paraffin, then sectioned at 5 μm. Sections were deparaffinised and stained with hematoxylin and eosin.

## Additional Information

**How to cite this article**: Khaydukov, E. V. *et al*. Riboflavin photoactivation by upconversion nanoparticles for cancer treatment. *Sci. Rep*. **6**, 35103; doi: 10.1038/srep35103 (2016).

## Supplementary Material

Supplementary Information
