# Supplementary material for: Riboflavin photoactivation by upconversion nanoparticles for cancer treatment
Source: Sci Rep. 2016 Oct 12;6:35103. doi: 10.1038/srep35103 (PMC5059683; doi:10.1038/srep35103)
Supplement: Supplementary Information [file srep35103-s1.pdf]

Supplementary Information

Riboflavin photoactivation by upconversion nanoparticles for cancer treatment

Authors:

E.V. Khaydukov[1*], K.E. Mironova[1,2*], V.A. Semchishen[1], A.N. Generalova[1,2], A.V. Nechaev[1,3], D.A. Khochenkov[1,4], E.V. Stepanova[4], O.I. Lebedev[6], A.V. Zvyagin[1,5†], S.M. Deyev[2†], V.Ya. Panchenko[1]

* First author/equal contribution

Affiliations:

[1] Federal Scientific Research Centre "Crystallography and Photonics" of Russian Academy of Sciences, Leninsky pr. 59, Moscow, 119333, Russia

[2] Shemyakin-Ovchinnikov Institute of Bioorganic Chemistry of the Russian Academy of Sciences, 16/10 Miklukho-Maklaya, Moscow 117997, Russia

[3] Institute of Fine Chemical Technologies, Moscow Technological University, 86 Prospect Vernadskogo, 119571 Moscow, Russia

[4] FSBSI "N.N. Blokhin Russian Cancer Research Center" of Ministry of Health of the Russian Federation, 24 Kashirskoye shosse, Moscow, 115478, Russia

[5] ARC Centre of Excellence for Nanoscale BioPhotonics, Macquarie University, North Ryde, NSW 2109, Australia

[6] Laboratoire CRISMAT, UMR6508, CNRS-ENSIACEN, Universite Caen, 14050 Caen, France

†Corresponding Author

A.V. Zvyagin (andrei.zvyagin@mq.edu.au),

S.M. Deyev (deyev@ibch.ru)

SI 1. Detection of singlet oxygen generated in the photosensitised riboflavin solution

In vitro assay of the singlet oxygen ($^1O_2$) production in aqueous solution of flavin mononucleotide (FMN) photosensitized by 405-nm light was carried out by using Singlet Oxygen Sensor Green (SOSG, Molecular Probes, US) molecular probe (Figure S1). Fluorimeter (Fluorolog-3, Horiba JY, France) was used for acquisition of the tested sample fluoresce, with a slip width set to 2 nm for both excitation and emission. SOSG reagent was dissolved in methanol to make a stock solution of 500 µM, and added to 100 µM FMN solution to the final concentration of 5-µM. The solution was prepared in a 5×5 mm quartz cuvette. SOSG fluorescence at 525 nm excited at 504 nm was markedly increased upon exposure to $^1O_2$ in the tested solution of FMN. In order to suppress the optical background due to a diode laser at 405 nm used for photosensitisation of FMN yielding $^1O_2$, a long-pass interference filter with a wavelength cut-off at 500 nm was inserted into the detection path of the fluorimeter. Sodium azide (NaN_3) was used as the quencher of $^1O_2$ to validate the assaying results.

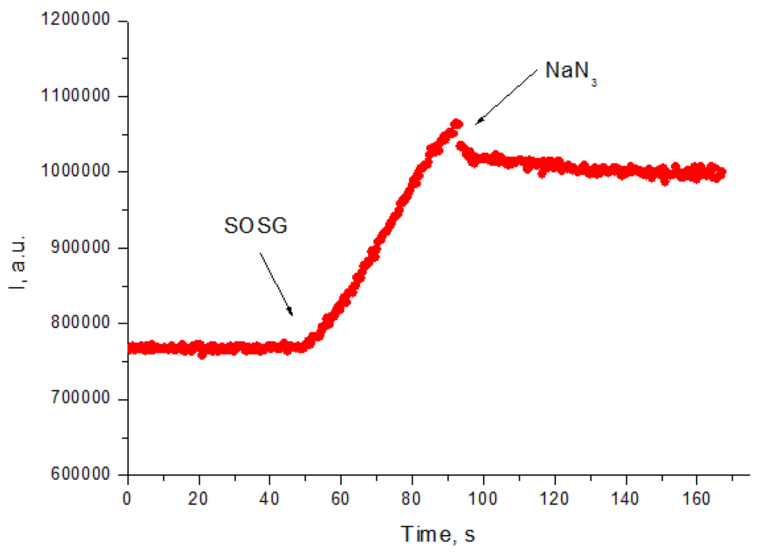

Figure S1. *In vitro* assay of the singlet oxygen ($^1O_2$) generation in FMN aqueous solution of the concentration 100 µM, using SOSG fluorescent molecular probe. A progressive increase of the SOSG fluorescence was observable at the start of the photosensitization process (marked by "SOSG") upon irradiation with 405-nm light. An addition of the inhibitor NaN_3 occurred at the time point "NaN_3" caused a quenching of the $^1O_2$ indicated by the plateau of fluorescence signal.

SI 2. Riboflavin uptake by cells measured by flow cytometry

Three types of cell lines were tested for the ability to accumulate riboflavin: Chinese Hamster Ovary (CHO) (Russian Cell Culture Collection), human breast adenocarcinoma SK-BR-3 (HTB-30™; ATCC) and primary human dermal fibroblasts, where the latter cells can be considered as a model of normal non-immortalised cells. The use of animal cell lines as negative controls for receptor-binding experiments is acceptable and broadly practiced, as it is evident from several selected citations [1-3]. An additional argument favoring CHO as the negative control is that it represented a highly aggressive cancer cell line with an anticipated high metabolic rate and hence high uptake level of Rf. Both cancerous and normal cells were incubated for 90 min in basal growth medium (McCoys 5A, HyClone, USA), which contained ~0.5-µM riboflavin. 2-µM

and 30-µM solutions of riboflavin (Rf) were added to the tested cells and no additional riboflavin was added to the control cells. The cells were washed twice with PBS at 4°C to arrest active cellular processes and assayed using a flow cytometer (Accuri C6 flow cytometer, BD, Oxford, UK), where an argon laser operating at wavelength of 488 nm was used as the excitation source, with the detection spectral range from 500 nm to 560 nm. The excitation wavelength fell into the excitation band of Rf (c.f. Figure 1b), whereas the detection band matched the fluorescence band of Rf. The intensity of riboflavin fluorescence was expressed as a median of the fluorescence intensity of 10000 cells. The cell assaying results are presented in Figure S2. In order to mitigate the nonlinear dependence of the fluorescence intensity versus the intracellular fluorophore concentration affected by the cell-specific microenvironment, the fluorescence intensity in tested cells was benchmarked and normalised with respect to that of the control cells, where endogenous riboflavin and its derivatives displayed measurable riboflavin fluorescence signal. In other words, Rf fluorescence intensity of the cells incubated in the basal growth medium was assigned a unity value. The Rf uptake of the investigated SK-BR-3 cells was ~3.5- and 1.5-fold of that of the CHO cells and fibroblasts, respectively, at the highest tested Rf concentration of 30 µM.

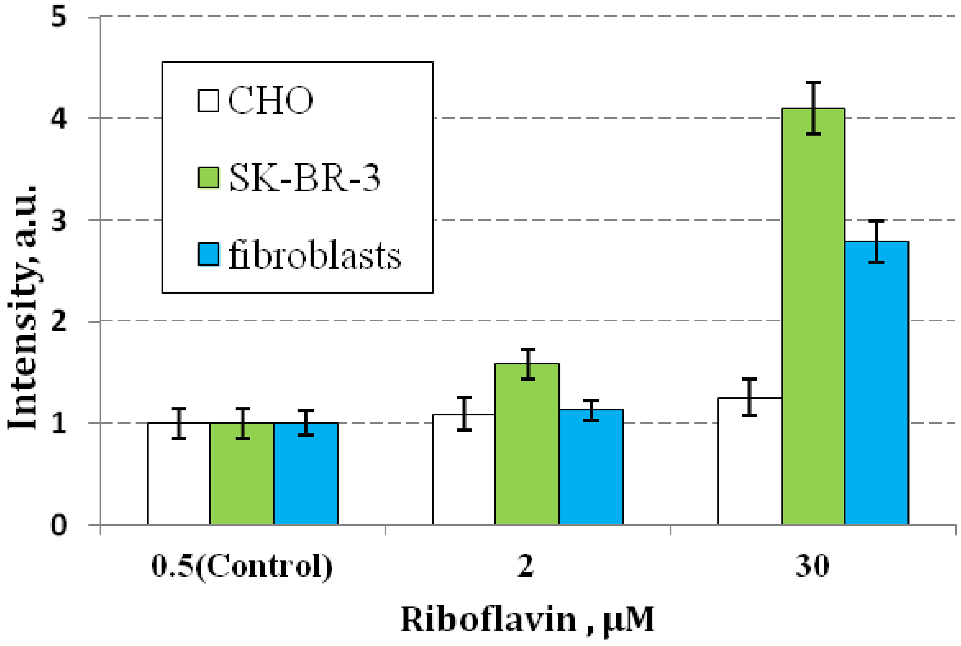

Figure S2 Fluorescence intensity of Rf (measured as a median of the fluorescence intensity of 10000 cells) at three concentrations of Rf in the growth media: 0.5 µM (control), 2-µM and 30-µM, indicating the accumulation of Rf in three cell lines: CHO, SK-BR-3 and fibroblasts presented as empty, green and blue bars, respectively.

SI 3. Cytotoxicity mechanism of photo-activated riboflavin

In order to pinpoint the PDT-induced death mechanism of cells pre-incubated with Rf, we carried out an apoptosis activity assay by using a flow cytometry system for different time points and measured the levels of active Caspase-3 by means of an immunofluorescence assay.

A HER2-positive cell line SK-BR-3 (HTB-30™) (ATCC) was used to figure out the main cytotoxicity mechanism at play when SK-BR-3 cells preincubated with flavin mononucleotide (FMN) were exposed to blue light radiation.

The cell line was cultured in culture medium RPMI-1640 (Gibco), containing 10% Fetal Bovine Serum (FBS) (HyClone); 2 mM/mL glutamine, 50 mg/mL penicillin-streptomycin (Gibco), at 37°C in atmosphere of 5% CO_2. This cell culture was maintained in the logarithmic growth phase by overloading the culture every 3-4 days. In order to perform assaying, cells were seeded in 6-well plates (Nunc) in concentration of 1×10^5/mL, and incubated for 24 h until a 70% monolayer was formed in complete RPMI-1640 medium. In order to assay mechanism of the phototoxic effect, cells were incubated in RPMI-1640 medium containing 30-μM of FMN for 90 min in the dark, then washed twice with RPMI-1640, and then irradiated with a diode laser at wavelength 450 nm for 10 min by setting the irradiation dose (I_{ex}) at 5.0 J/cm^2 . The exposed cells were cultured in RPMI-1640 with 1% FBS for 6, 12, 24 hours, then washed twice with ice-cooled PBS and lifted from the well plates using TrypLE Select (Thermo). SKBR-3 cells incubated in RPMI-1640 with 1% FBS and 40-μM Etoposide were used as the positive control, where Etoposide served as an inhibitor of Topoisomerase II, which is known as an active promoter of apoptosis. The other employed controls were, as follows: cells exposed only to radiation at 450 nm for 10 min at I_{ex} = 5.0 J/cm^2 (no preincubation with FMN); cells incubated with FMN for 90 min (no light exposure); and just untreated cells, were used as three negative controls. The treated and control cell samples were immediately assayed using Muse Cell Analyzer (Millipore) with Muse® Annexin V and Dead Cell Assay Kit (Figure S3).

The result analysis demonstrated that exposed to 450-nm light in combination with the pre-incubation with FMN had a profound cytotoxic effect on SKBR-3 cells in contrast with the separate treatment with light or FMN.

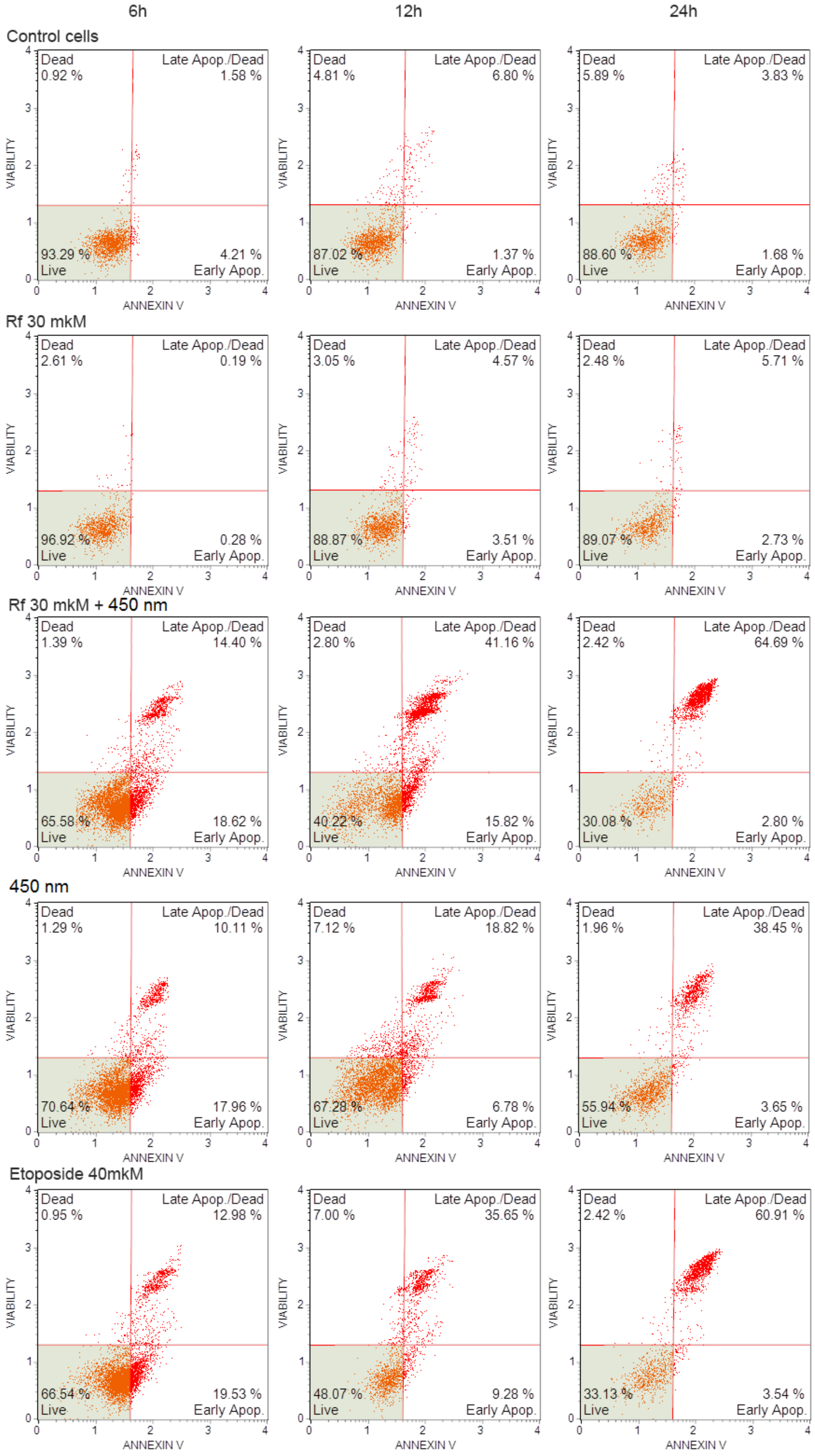

Figure S3. Cell viability and apoptosis assay of SKBR-3 cells treated with FMN and exposed to 450-nm light radiation. A top right quadrant was used for detection of apoptotic cells.

In order to determine the main mechanism accountable for the reported SK-BR-3 cells death, we assayed the levels of activie of Caspase-3 in the treated cell samples and controls. SK- BR-3 cells were seeded in 4-well culture slides (BD Falcon), concentration 5×10^4/mL and incubated for 24 h at 37 ^{0}C in atmosphere 5% CO_2. As described above, cells were incubated with 30-µM FMN for 90 min and exposed to 450-nm light radiation for 10 min. The positive (Etoposide 40-µM) and negative controls were prepared following the protocols described above. Caspase-3 assaying was carried out by immunocytochemistry staining 6, 12 and 24 h after the cell treatments. Briefly, slides with cell were washed once in PBS, then fixed with ethanol and acetone, incubate with blocking solution (Dako) and permeabilised using 0.1-M citrate buffer with 0.1% Triton-X100 for 5 min on ice. Cells were then stained with FITC-conjugated anti-active caspase-3 mAb (BD) for 60 min at RT in the dark. Cells were washed twice in PBS and stained with Hoechst 33258 dye (Sigma), following the incubation, and then analysed with InCell Analyzer 6000 (GE Healthcare).

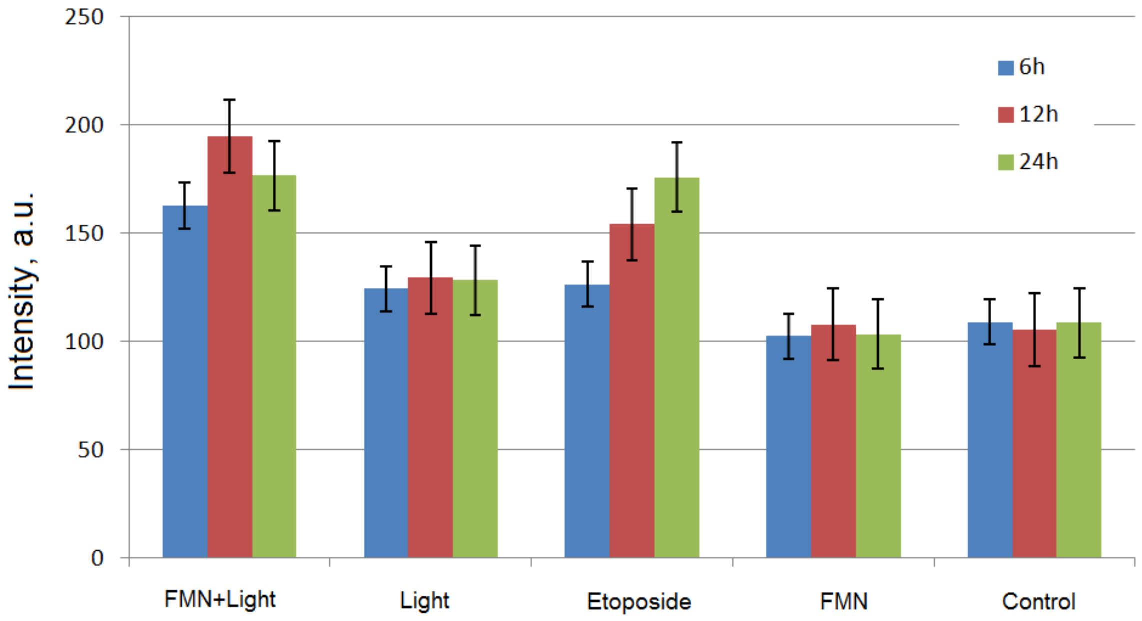

Figure S4. A histogram of the active Caspase-3 expression in 450-nm light treated SK-BR-3 cells preincubated with FMN (Riboflavin) and in the positive (Etoposide 40 µM) and negative (450 nm light, FMN, Control) controls.

Fluorescence laser-scanning confocal microscopy images of immunocytochemistry-stained SKBR-3 cells 12-h post-treatment with the 450-nm laser radiation is shown in Figure S5. The primary antibody of the staining was raised against active Caspase-3.

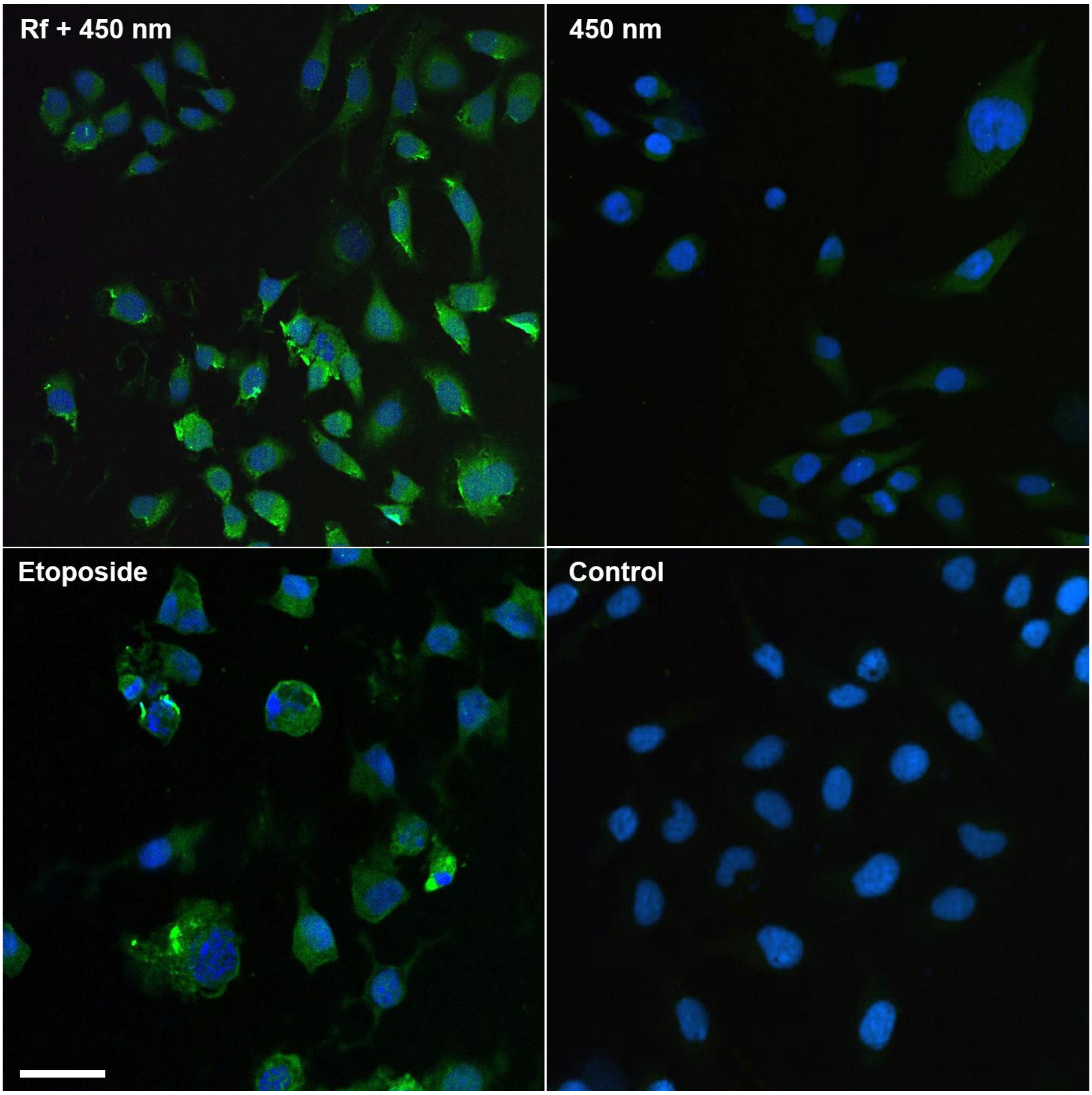

Figure S5. Fluorescence confocal microscopy images of the expression of active Caspase-3 in SKBR-3 after 12 h of the cell treatment. Green and blue colours show fluorescence signals from FITC-conjugated anti-active caspase-3 mAb and Hoechst, respectively, which report on the Caspase-3 activity and morphology of cell nuclei. Scale bar 50 µm.

Based on result analysis shown in Figures S3, S4 and S5, we can infer that SK-BR-3 cells treated with FMN and exposed to 450-nm light radiation underwent apoptosis, which was manifested by the active expression of Caspase-3. At the same time, neither FMN, nor 450-nm light radiation treatment, nor treatment-free induced significant expression of Caspase-3. An increase of Caspase-3 was notable in the case of 450-nm light radiation treatment, although the effect was marginal. In summary, the positive result of the Caspase-3 assaying in FMN+blue light treated SK-BR-3 cells indicated the apoptotic cytotoxic pathway via Caspase-3 activation.

SI 4. Epi-luminescence imaging system

We designed and set up a two-channel epi-luminescence imaging system, its simplified schematic diagram is shown in Figure S6 (left panel). A pencil-shaped collimated cw semiconductor laser beams (beam diameter, 2 mm) at wavelengths of 975 and 450 nm were raster-scanned by the fast galvanometer scanning mirror units (Miniscan-07, Raylase, Germany) across a laboratory animal, and the excited photoluminescence was collected from the same side with a camera lens (f-number 0.95). The 450-nm and 975-nm laser excitation beams were used to image FMN and UCNPs distribution in a mouse (c.f. Figure 1f and Figure 3a). The excited fluorescence and photoluminescence (PL) signals were recorded by an electron-multiplication (EM) CCD camera (Falcon, Raptor Photonics, Ireland). The camera lens was adjusted to optically conjugate the EMCCD camera sensor plane and the mouse dorsal side. Interference filters (Semrock, USA) blocking both excitation radiations were used in the detection path in front of the EMCCD.

The comparison of the image acquisition in the Stokes and anti-Stokes channels shows much greater contrast in the anti-Stokes channel. This clearly demonstrates the advantage of UCNP-assisted imaging, as reported in many publications [4]. The fluorescent image in the Stokes channel was overshadowed by the live tissue autofluorescence.

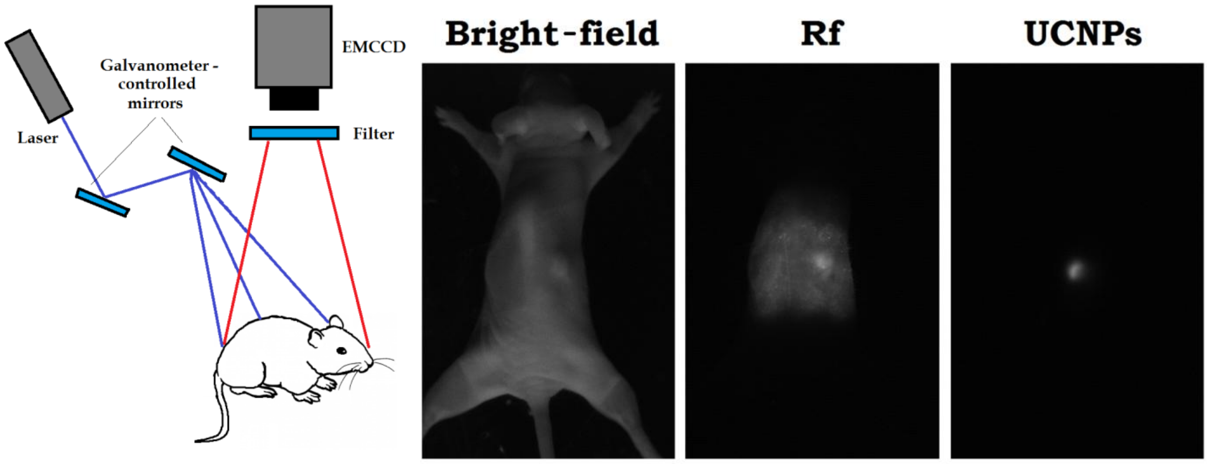

Figure S6. Left panel, a simplified schematic diagram of the epi-luminescence imaging system. In vivo photoluminescence imaging of a live mouse, post 1.5-hour intratumoural injection of FMN+UCNP. Image panels: Bright-field, Rf and UCNPs display a bright-field image, FMN fluorescence image acquired upon the excitation with the 450-nm laser, i.e. in a Stokes channel, and PL image acquired upon the excitation with the 975-nm laser, i.e. in an anti-Stokes channel.

SI 5. Conversion efficiency measurements

Conversion efficiency denoted as η_{UC} represents one of the most important photoluminescence parameters [5] of UCNPs defined as the emitted power per absorbed power P_{em}/P_{abs} measured in W/W. The use of η_{UC} is inconsistent in the literature, where it is most often termed “quantum yield” (QY), which is properly defined as a ratio of the number of photons emitted to the number of photons absorbed [6]. In the upconversion process, however, one photon is emitted per two or more photons absorbed (c.f. Figure 2b), so the QY ≤ 50% according to this definition. In order to re-normalize this value, the QY of 2- or many- photon

absorption processes must be scaled [7] by an unknown factor resulting from the UCNP complex excitation pathways , which is recognized in literature [8]. In view of these reasons, we believe that the term "conversion efficiency" more adequately captures the nature of the upconversion processes and permits its correct expression [5].

The conversion efficiency η_{UC} measurements were performed using a calibrated integrating sphere setup, as described previously [8,9]. An integrating sphere allows accurate measurement of the absolute absorption and emission in virtue of its ability to spatially integrate the corresponding radiant fluxes to minimize the measurement artefacts due to the scattering by UCNP powder particles. UCNPs powder to be measured was placed in a sample holder and illuminated by a 975-nm laser light through an entrance port of the integrating sphere. Using appropriate filters to account for the UCNP spectrum (Figure S7a) and a photodiode placed at a perpendicular exit port of the sphere, both emitted P_{em} and absorbed P_{abs} powers were measured over a range of the excitation intensities as shown in Figure S5b. η_{UC} was calculated using the definition of $P_{em}\,/P_{abs}$ [W/W].

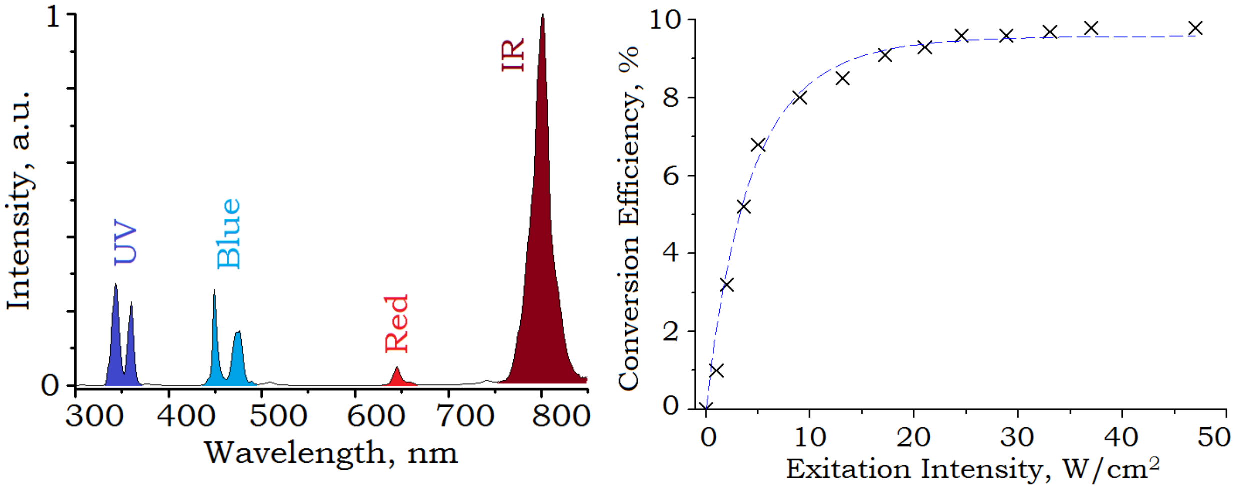

Figure S7. An emission spectrum of the UCNPs powder with the excitation by a 975-nm laser at the intensity value of 50 W/cm^2. Note strong UV and Blue spectral bands in comparison with the IR band. Absolute conversion efficiency of UCNPs versus the excitation intensity at 975 nm measured using the calibrated integrating sphere setup. The saturation was reached at ~25 W/cm^2. The blue dashed line is provided as a guide to the eye.

An emission spectrum of the UCNPs powder was acquired using a calibrated fluorimeter (Fluorolog-3, Horiba JY, France) with excitation by a 975-nm laser at the intensity 50 W/cm^2, and is shown in Figure S7a. The spectrum featured four emission bands (colour-coded in Figure S5a), which are known to result from the Tm^{3+} electronic transitions.

The saturation was reached at the relatively low value of η_{UC} ~25 W/cm^2, which was advantageous for optical imaging at the deeper layers in biological tissue. Indeed, as pointed by Vinegoni et al. [10], since the excitation intensity decays as $1/d^2$ (*d* – depth in highly scattering biological tissue medium), the excitation efficiency drops to at least $1/d^4$ assuming two-photon absorption process, and therefore it becomes critically important to maintain the lowest possible saturation intensities of UCNPs. Chen et al. have demonstrated imaging at the depth of 3 cm in adipose tissue by using specifically produced UCNPs with as low saturation intensity [11]

as ~10 W/cm^2. The maximum achievable value of the η_{UC} at I_{ex} ~50 W/cm^2 is estimated as ~10%, which represents one of the highest values of η_{UC} in comparison with that reported by Zhao et al. [9].

SI 6. Investigation of the photon energy transfer processes: from donor UCNP to acceptor flavin mononucleotide (FMN)

Figure S8 shows emission spectra of core/shell $NaYF_4$:Yb^{3+}:Tm^{3+}/ $NaYF_4$ nanoparticles in aqueous solution under the excitation with 975-nm light without and with the presence of FMN. The red (650 nm $^1G_4 \rightarrow {}^3F_4$) spectral band of thulium was used as a reference to normalize the acquired spectra. UV and blue thulium emission bands at 345 ($^1I_6 \rightarrow {}^3F_4$), 360 ($^1D_2 \rightarrow {}^3H_6$), 450 ($^1D_2 \rightarrow {}^3F_4$) and 475 nm ($^1G_4 \rightarrow {}^3H_6$) effectively contributed to the excitation of the FMN acceptor, which was manifested by its emission in a green spectral band peaked at 540 nm, and by the $^1O_2$ generation (c.f. Figure 1a). It is difficult to determine whether the UV-blue band depletion was due to the Föster (FRET) or light (LRET) resonance energy transfer processes by analyzing Figure S8. The emission band depletion spectral dependence matched that of the Rf absorption band. The saturation dependence of the UCNPs+FMN solutions versus FMN concentration pointed to the dominant contribution of the LRET processes. Indeed, at the Rf concentration of 0.22 mg/mL, the photon mean free path [$l_{Rf} = 1/\alpha_{Rf} = 1/(\varepsilon_{Rf} \times C_{Rf})$], where α_{Rf}, ε_{Rf}, C_{Rf} denote linear absorption coefficient, molar extinction coefficient and molar concentration of Rf, respectively, becomes comparable to the cuvette linear dimension of 0.5 cm, so that UV-blue photons emitted by UCNPs in the colloidal suspension are highly likely to be absorbed by Rf molecules.

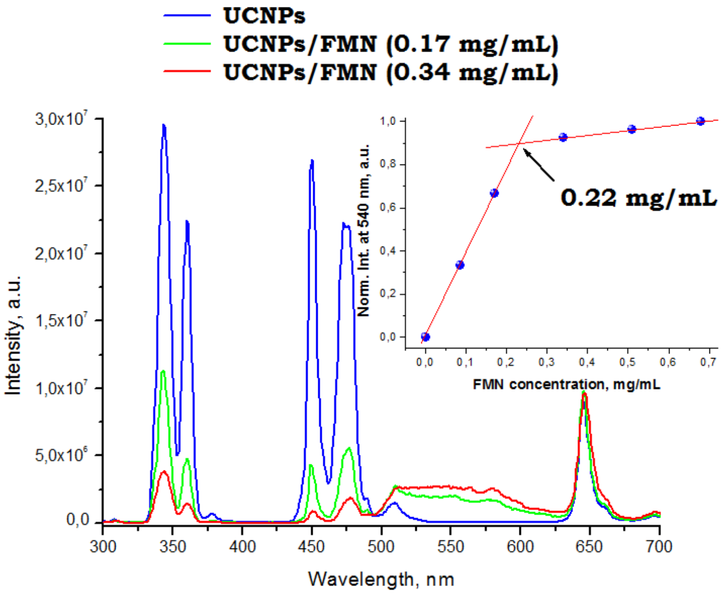

Figure S8. Emission spectra of aqueous colloidal core/shell $NaYF_4$: Yb^{3+}:Tm^{3+}/ $NaYF_4$ nanoparticles under the excitation with 975-nm light without (blue curve) and with 0.17 and 0.34 mg/mL, (green and red curves, respectively) FMN added to the aqueous solution. Inset: A plot of the normalized FMN photoluminescence intensity at 540 nm versus FMN concentration in the aqueous solution of UCNPs-FMN. The PL saturation concentration of FMN was measured to be 0.22 mg/mL.

In order to detect whether FRET process had a measurable contribution to the detected photon energy transfer, we performed measurement of the UCNPs lifetimesτ_{UP}. It was expected that τ_{UP} decreased in the presence of acceptor, as it provided an additional channel of the excited state relaxation. The FRET-induced decrease of the donor lifetime is regarded as one of the most reliable hallmarks of the FRET process.

τ_{UP} -s of the Tm^{3+} transitions $^1G_4 \rightarrow {}^3H_6$ (475 nm), $^1D_2 \rightarrow {}^3F_4$ (450 nm), $^1D_2 \rightarrow {}^3H_6$ (360 nm) and $^1I_6 \rightarrow {}^3F_4$ (345 nm) were measured without (green) or with (red) the presence of an acceptor FMN, the results presented in Figure S9. The decay time of $^1G_4 \rightarrow {}^3H_6$ (475 nm) and $^1D_2 \rightarrow {}^3H_6$ (360 nm) in the presence of acceptor changed from 645 ± 5 µs to 575 ± 5 µs and from 317 ± 4 µs to 310 ± 3 µs, respectively. τ_{UP} -s of the transitions $^1D_2 \rightarrow {}^3F_4$ (450 nm) and $^1I_6 \rightarrow {}^3F_4$ (345 nm) were measured as 319 ± 4 µs and 238 ± 4 µs, respectively, with a negligible variation within the measurement error. We conclude that two transitions $^1G_4 \rightarrow {}^3H_6$ and $^1D_2 \rightarrow {}^3H_6$ were involved in the detected FRET processes. We speculate that these transitions were dominant over the other two transitions due to their relaxation to the ground state.

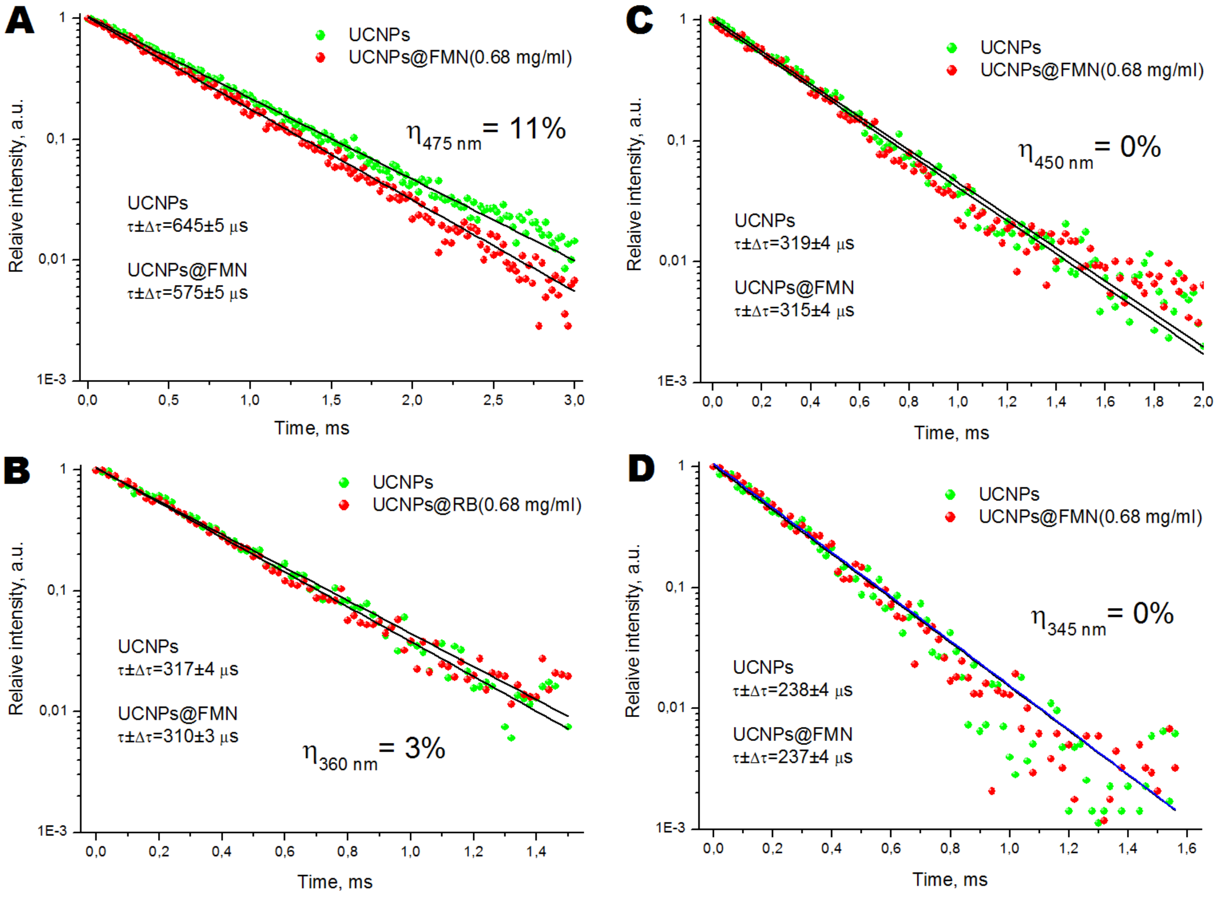

Figure S9. Photoluminescence decays (normalized) of the UCNP donor transitions without (green) or with (red) the presence of an acceptor FMN: A - transition $^1G_4 \rightarrow {}^3H_6$ (475 nm), B - transition $^1D_2 \rightarrow {}^3H_6$ (360 nm), C - transition $^1D_2 \rightarrow {}^3F_4$ (450 nm) and D - transition $^1I_6 \rightarrow {}^3F_4$ (345 nm).

The efficiency of the FRET process was found from following equation:

$$\eta_{UP} = \left(1 - \tau'_{UP}/\tau_{UP}\right), \qquad (1)$$

Were τ_{UP} is the decay time of the excited state of donor (UCNP) and $\tau_{UP}^{'}$ is the decay time of the excited state of the donor in the presence of the acceptor (FMN). According to the decay measurements, the energy transfer efficiencies of the $^1G_4 \rightarrow {}^3H_6$ (475 nm) and $^1D_2 \rightarrow {}^3H_6$ (360 nm) transitions were found to be 11 % and 3 %, respectively.

SI 7. Evaluation of the FRET process in UCNPs coated with PMAO/FMN

Among several approaches of realizing FRET, including the FRET process as discussed in the main body of the paper, we explored a possibility to impregnate riboflavin molecules into a polymeric shell, which was used for surface functionalization of UCNPs. Amphiphilic polymer, poly(maleic anhydride-*alt*-1-octadecene) (PMAO) was used as the polymeric shell previously reported in [12]. Oleic functional groups on the surface of an as-synthesised UCNP provided anchoring points for hydrophobic terminals of the amphiphilic polymer. The hydrophilic terminals of the amphiphilic polymer became directed outwards (i.e. into aqueous environment) rendering UCNP hydrophilic.

The PMAO surface-coating procedure based on a solvent evaporation method has been detailed elsewhere [12,13]. In brief, UCNPs were dispersed in chloroform, and then polymer solution in solvent miscible with chloroform was added dropwise to aqueous solution taken in at least tenfold excess, following ultrasonic treatment and stirring of the mixture. As a result, stable colloids of UCNP-PMAO in the aqueous solution were produced.

The embedment of riboflavin molecules into the polymer layer of the UCNP-PMAO was realized by inducing the layer swelling during incubation with riboflavin to enable its entrapment in the swollen polymer layer. In order to realize this approach, we used the following protocol. 0.5 mL of UCNP-PMAO aqueous colloid (0.5 mg/mL) was centrifuged at 10,000 rpm for 10 min, then the pellet was dispersed in a mixture of Na-phosphate buffer, pH 6.0 and ethanol with a ratio 9:1, and incubated at room temperature for 30 min to allow swelling of the PMAO shell on the UCNP surface. Then an aliquot of 0.3 mL 0.15 % wt. (0.45 mg) aqueous solution of Rf was added and the reaction tube was blanketed in a foil. The mixture was sonicated for 10 min and incubated for 1 h, while stirring at room temperature. To remove an excess of Rf, the mixture was washed three times with water (consecutive centrifugation at 10000 rpm for 10 min – re-dispersion steps). The pellet was then dispersed in 0.5-mL PBS buffer pH 7.0.

The riboflavin concentration in the resultant nanocomplex UCNPs-PMAO/Rf was evaluated as a difference in the concentrations of the added and unreacted Rf. The unreacted Rf was assayed in the supernatant after the centrifugations by means of a spectrophotometer Evolution 201 (Thermo Scientific, Finland) versus PBS buffer pH 7.0 at λ = 267 nm. The results obtained in absorbance units (optical density) were represented in terms of the Rf concentrations using a calibration graph of the absorbance of pre-measured concentrations of Rf dissolved in PBS buffer pH 7.0. As a result, we evaluated the Rf content as 0.06 mg in 0.5 ml PMAO-modified UCNPs (0.5 mg/ml).

Thus, we designed and produced a resonance energy transfer nanocomplex comprising the donor (UCNP-PMAO) and acceptor (Rf). A spectrum of this nanocomplex dispersed in PBS

buffer (pH 7.0) is presented in Figure S10. One can see a fluorescence band from 500 nm to 620 nm acquired with the excitation by a 975-nm laser (green-coloured), which we ascribe to Rf (also, compare with Figure 1b). The fluorescence band of Rf was delineated from the UCNP emission spectrum to be comparative recording and analysis of UCNP-PMAO and UCNP-PMAO/Rf spectra. The emergence of the Rf fluorescence signal upon the 975-nm excitation was a clear indication of the energy transfer between UCNP and Rf under 975 nm excitation in a respective configuration "donor-acceptor".

In order to determine partial contribution of FRET and LRET processes to the resonant energy transfer process, we recorded and analyzed the UCNPs (donor) emission decay lifetimes (τ_{UP}), as it was described in Section **SI 6** of Supplementary Information. No changes of τ_{UP} was detected across the UCNP-PMAO and UCNP-PMAO/Rf samples. Based on this result, we conclude that LRET was a dominant contributor to the resonant energy transfer process.

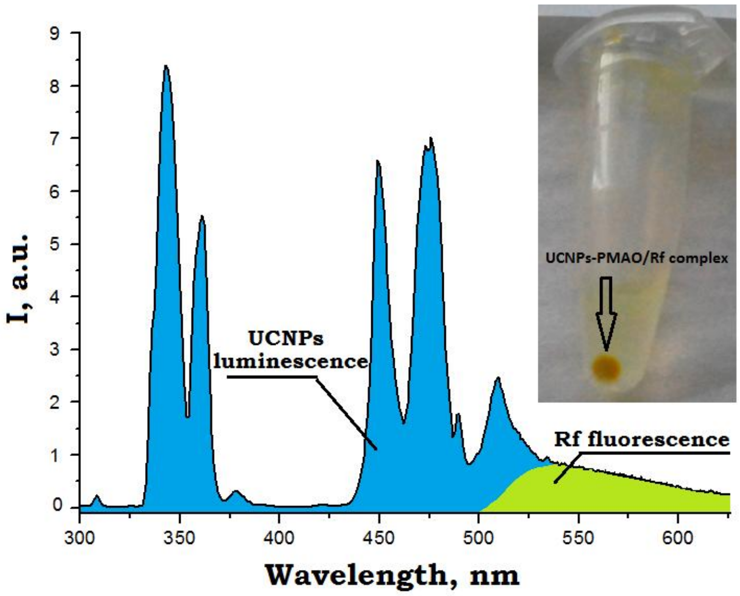

Figure S10. Photoluminescence spectrum of UCNP-PMAO/Rf complex under 975 nm excitation and the sample of UCNPs-PMAO/Rf complex after centrifugation.

This result enables us to comment on a number of the reported FRET results with UCNPs as the donor, for example [14]. It seems that the authors observed LRET processes and termed these "FRET". Firstly, no comparative analysis of the τ_{UP} of the donor (UCNP) and donor-acceptor (UCNP-acceptor) was presented, which would represent the most convincing evidence of the FRET process. Secondly, the UCNP emission takes place in the core and subsurface region of the UCNP nanoparticle, where only subsurface region is "FRET-active". This is exacerbated by the necessity to mount a shell layer on the active volume of the nanoparticle (core/shell architecture) to seclude it from emission-quenching environment. This leaves only a fraction of activator ions to participate in the FRET process. In two cases presented in this work, the UCNPs-PMAO/FMN nanocomplex seemed to support LRET process with a negligible FRET contribution. Indeed, the active UCNP core was separated from FMN acceptors by a passive shell of $NaYF_4$ and amphiphilic polymer (PMAO) coating resulting in a donor-acceptor distance separation of >10 nm, which effectively precluded FRET. In case of the FRET-optimised

configuration, i.e. bare UCNP (UCNP-TMAH, see Figure 2) with riboflavin molecules interacting dynamically, the FRET contribution was evaluated to be ~14 %. This is still important for implementation of nanometer-scale localized photoreactions, although LRET remained the main contributor to the overall efficiency of the photo processes described in this work.

SI 8. Laser-illuminated wide-field epi-luminescent microscope

A diode laser (ATC-C4000-200-AMF-975, Semiconductor devices, Russia) operating at wavelength 975 nm delivered the excitation light to the sample plane of an inverted epi-luminescence microscope (Motic AE30-31, China). The excitation beam was filtered with a laser-line filter and shaped to converge at the back focal plane of a long-working distance objective lens (40×, NA 0.5, Motic, China), which was used for both bright-field and photoluminescent-field cell imaging. This Köler-type laser illumination provided uniform illumination of the sample plane, as detailed elsewhere [5]. The sample plane was imaged using an 4.2-megapixels sCMOS CCD camera (Gen II, Photonic Science, GB). SK-BR-3 cells grown in a 96-well plate were used as the specimens for most of the described optical microscopy procedures. A micro-thermocouple (76 µm, Omega Engineering, USA) was employed for temperature control of a culture medium, which was maintained at < 32.5 °C during the cell exposure to the laser irradiation, including PDT procedures.

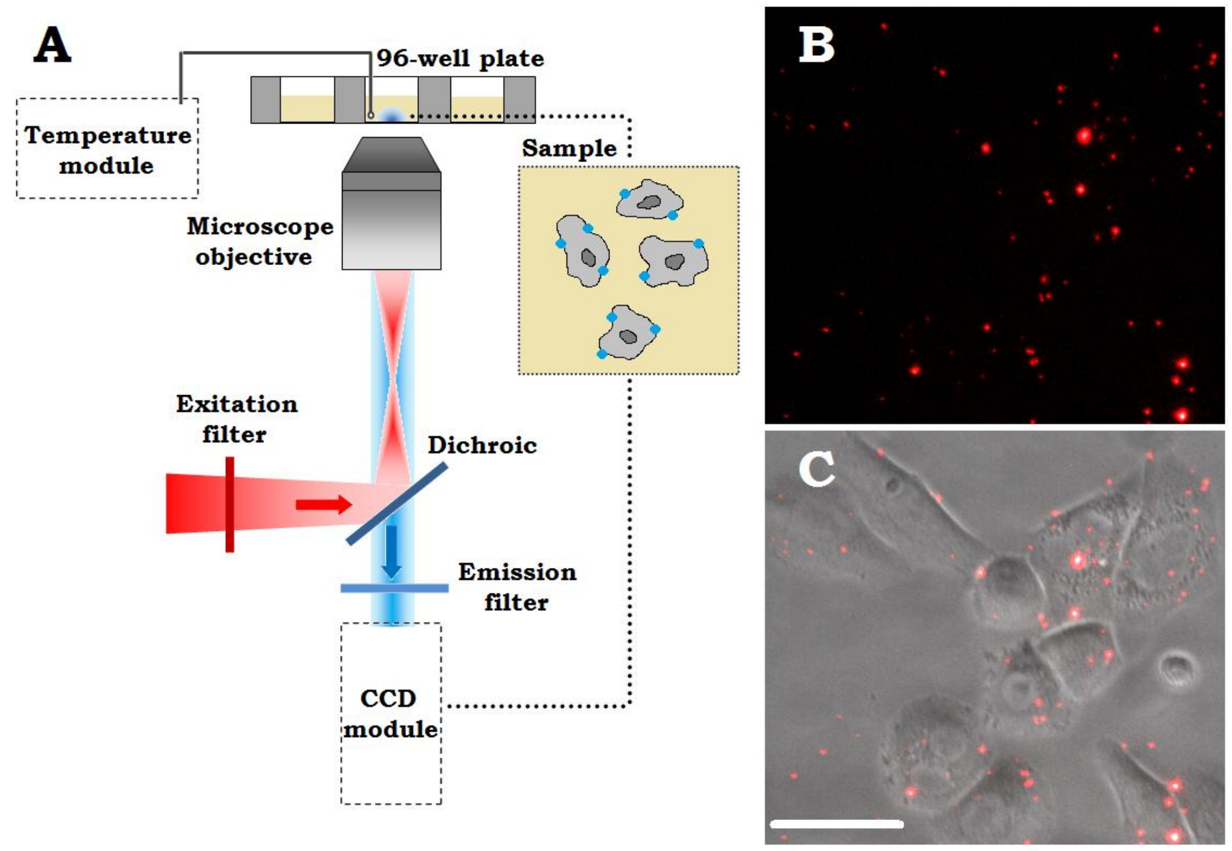

Figure S11. (A) A schematic diagram of a cell imaging setup. Examples of the epi-luminescence microscopy images of SK-BR-3 cells non-specifically labeled with TMAH surface-modified UCNPs: (B) a photoluminescence image; (C) an overlay of the photoluminescence and bright-field images (c). Scale bar, 30 μm.

SI 9. Near-infrared photodynamic therapy of cells mediated by riboflavin mononucleotide (FMN) sensitised by upconversion nanoparticles (UCNPs)

Near-infrared phototoxic action of UCNP/FMN pair was demonstrated on SK-BR-3 cells *in vitro* by using the following procedure. SK-BR-3 cells grown to full confluency were incubated at +37 °C in a CO_2-incubator (5 % CO_2) for 24-h in culture medium (McCoys 5A, HyClone, USA). Then the culture medium was removed and UCNPs surface-modified with tetramethyl-ammonium hydroxide (TMAH-UCNPs) in PBS solution (~40 µg/mL) were added to the cells. The solution was removed after incubation for 5 min, followed by two washing cycles with PBS. Then 100-µM FMN solution in PBS was added in light-dimmed environment, immediately followed by the sample irradiation with a 975-nm laser light to achieve irradiation dose of 600 J/cm^2. Three different control experiments were performed: 1) incubation of the SK-BR-3 cells with TMAH-UCNPs only, as described above, followed by the same irradiation dose; 2) incubation of the SK-BR-3 cells with TMAH-UCNPs, followed by addition of the FMN solution, as described above, without the irradiation; and 3) incubation of the SK-BR-3 cells with the FMN solution, followed by the same irradiation dose of 600 J/cm^2. The results are presented in the main text clearly showing the combined phototoxic effect of the UCNP-FMN pair.

We wish to note an important observation. The phototoxic efficiency of UCNP-FMN pair was found maximum at the incubation time of TMAH-UCNP in SK-BR-3 cell cultures of 5 min. Passing this time point, the efficiency of the phototoxic action of UCNP-FMN pair decreased dramatically versus the incubation time. Since it is well-known that such short incubation time duration sufficed only for binding our nanoparticles to the cell membrane, we hypothesize that the phototoxic mechanism was localised to the cell membrane. The phototoxic effect maximized at the comparable incubation times has been reported in literature, where it has been consented that the most probable scenario involved generation of singlet oxygen characterized by the short-range action (hundreds of nanometres), and hence related to the cell membrane permeabilisation, leading to crucial disruption of the cell functionality and eventually, cell death.

The experimentally measured threshold irradiation dose of 600 J/cm^2 sufficed to induce the cells death was dependent on the concentration of UCNPs immobilised on the cell membranes. This threshold increased, as the UCNP concentration decreased.

In addition, although UV radiation photosensitised by our UCNPs entailed cytotoxic effects, the cytotoxicity measured in terms of IC50 was at least ten-fold lower than that induced by the combined action of UCNP-FMN, and hence was omitted from the present study.

SI 10. Near-infrared photodynamic therapy (PDT) mediated by FMN sensitised by UCNPs in laboratory animals *in vivo*

A diode laser (ATC-C4000-200-AMF-975, Semiconductor devices, Russia) operating at 975 nm in a pulsed regime (pulse width 50 ms, repetition rate 8 Hz) was employed for *in vivo* PDT treatments. Laser light was coupled to a multimode optical fibre probe for flexible delivery to tumour sites. Figure S12 shows tumour irradiation with this fibre optical probe. An SK-BR-3

xenograft-bearing mouse was fixed on a plate by using a duct tape. The distance between the tumour and fibre probe was set to limit the average laser intensity at the level of ~ 1.5 - 2 W/cm^2 to prevent overheating of the mouse tumour and surrounding tissue. A tumour site was irradiated in a raster-scanning regime with a dose 900 J/cm^2 at 975 nm, following UCNPs+FMN peritumoural injection and incubation for 1.5 h. The tumour site temperature was monitored by means of a FLIR A655sc Infrared Camera (FLIR, USA) to ensure the safe physiological margins of the procedure (< 41 ^{o}C).

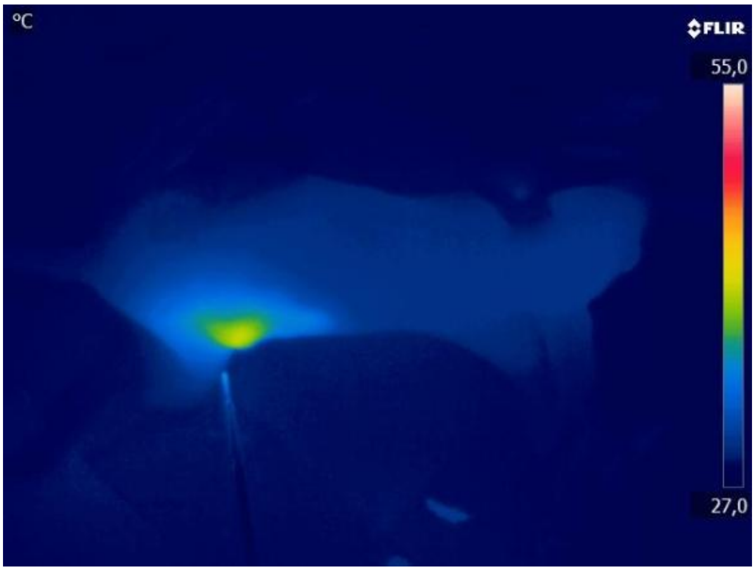

Figure S12. Temperature image of an immunodeficient mouse bearing a grafted SK-BR-3 tumour during the PDT treatment being irradiated with a 975-nm laser coupled to a multimode optical fibre operating at the intensity of 1.5 - 2 W/cm^2.

SI 11. Possible mechanisms of the observed high-efficiency near-infrared PDT

The observed near-infrared PDT treatment outcome appeared to be remarkable and hence worth commenting. One of the possible PDT action mechanisms was tumour-associated vascular damage, leading to thrombosis and haemorrhage, which subsequently caused the tumour hypoxia, as reported in Refs. [15-17]. The analysis of the histology and immunocytochemistry results of the excised PDT-treated SK-BR-3 cancer tissue revealed severe damages of the tumour blood vessels, with extensive hemorrhages (c.f. Figure 3d). This caused the vast elimination of tumour cells from the vicinity of capillaries. The PDT-induced haemorrhage and occlusion of the capillaries led to concomitant hypoxic events in the remote areas of the tumour tissue that caused the cessation of its growth and eventually tumour remission.

In order to confirm that the successful PDT effect was due to the coalescence of the main PDT components, UCNPs, FMN and 975-nm laser irradiation, we performed a number of control experiments, presented in Figure S13. The tumour regression effect was apparent and statistically significant only when all three treatment components were used (shown in red). The experimentally determined threshold irradiation dose of 600 J/cm^2 in the cell experiments

was increased to 900 J/cm^2 in the animal PDT experiments to account for the extinction effect due to the scattering and absorption effect of live biological tissue.

We also note that in contrast with the observed PDT effects, cancer cells remained viable in the tumour tissues featuring no noticeable disorders and symptoms indicating the tumour regression, in cases of administering UCNPs+FMN, without the laser-treatment and 975-nm laser irradiation without UCNPs+FMN injection.

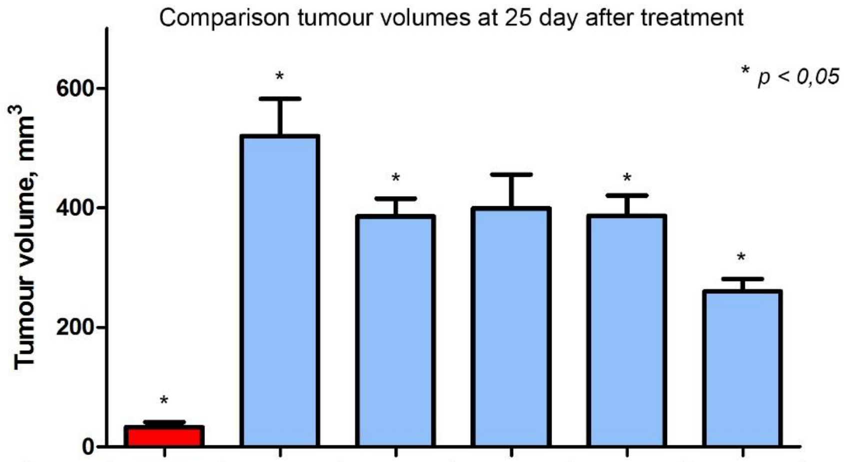

UCNPs	+	−	+	−	+	+
FMN	+	−	+	−	−	−
Laser 975 nm	+	−	−	+	−	+

Figure S13. Histogram of the SK-BR-3 tumour xenograft volume on the day 25 following treatment versus the following main treatment components: peritumoural injection of our TMAH-surface-modified upconversion nanoparticles (UCNPs), flavin mononucleotide (FMN); and irradiation with the 975-nm laser of the therapeutic dose of 900 J/cm^2. A table below the histogram displays the combination of the components, with "+", "-" indicating "using", "not using" the component in the procedure. Statistical significance $p < 0.05$.

SI 12. Near-infrared PDT mediated by FMN sensitised by UCNPs in laboratory animals *in vivo*

The demonstrated ability of the near-infrared excitation light at 975 nm to penetrate the whole cancer lesion in the tested immunodeficient mice (as described in Figure 3) to photosensitise UCNP/FMN PDT agents was surprising. It is recognised that the excitation intensity is attenuated as $I_{ex} \sim 1/d^2$ (d – depth in biological tissue), whereas PL signal is proportional to I_{ex}^n , where n varies approximately from 2 to 5 for the near IR and UV emission bands, respectively (c.f. Figure S7), in the non-saturated regime. Hence, one can expect a dramatic drop in the intensity of the UV-blue band of the UCNP emission, when these nanoparticles are buried in biological tissue, such as a tumour xenograft. Aiming to illustrate (rather than quantify) that the demonstrated in the PDT experiment FMN photoactivation was possible via the energy transfer from the UV-blue band of UCNPs in biological tissue, we designed and fabricated a biological phantom, which reproduced the key optical properties of the biological tissue in the near-IR spectral range. This biological tissue phantom was made using polyvinylchloride plastisol (M-F Manufacturing Co., USA) by a method described in [18], with

an admixture of high-refractive index TiO_2 nanoparticles (Sigma-Aldrich, Germany) to model the pre-calculated scattering properties of the biological tissue. The scattering, absorption coefficient and anisotropy factor were measured as μ_s = 35 cm^{-1}, μ_a = 0.5 cm^{-1} and g = 0.57 in the near-IR spectral range, respectively. As-synthesised UCNPs (described in M&M) were homogeneously embedded using ~2 % volume fraction in a 1-mm thin polymer film, which was mounted on a glass substrate. The excitation of 975-nm of the intensity level was comparable to that used in the *in vivo* PDT experiment, i.e. < 2 W/cm^2. Figure S14 shows a schematic diagram of the experimental set up used for the demonstration. As it is apparent in Figure S12, visible intense blue light was emerging from the UCNP film upon excitation with the 975-nm laser through the 4-mm-thick biological tissue phantom. This intensity of the blue light was visually comparable with that used in the measurement of $^1O_2$ production in the UCNPs+FMN colloid (c.f. Figure S8), and provides qualitative assurance of the feasibility of the demonstrated PDT photosensitised by the UCNP-FMN.

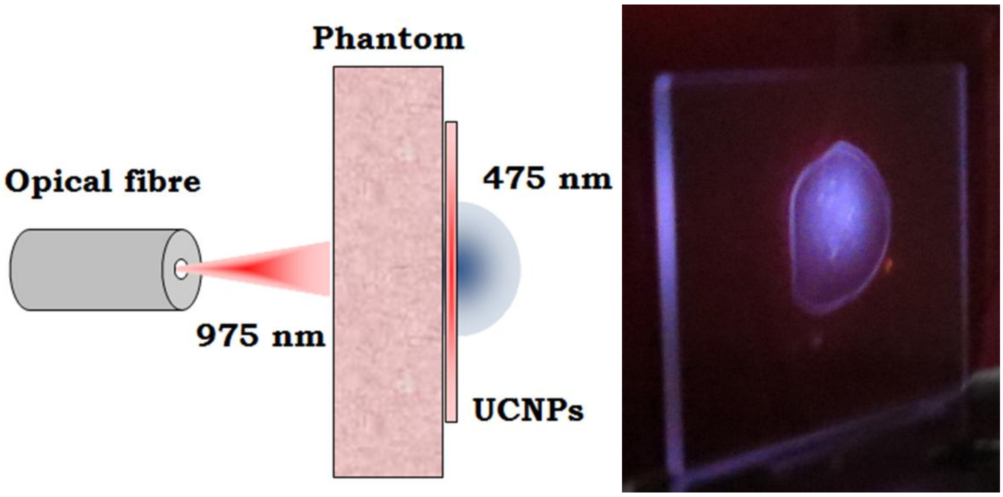

Figure S14. A schematic diagram of the experimental modelling of the excitation of an UCNP sample placed to the right-hand side a biological tissue phantom. Blue photoluminescence signal from the UCNP sample immersed in the phantom to a depth of 4 mm under 975 nm excitation was demonstrated.

References

1 Hathaway, H. J. *et al.* Detection of breast cancer cells using targeted magnetic nanoparticles and ultra-sensitive magnetic field sensors. *Breast Cancer Research* **13** (2011).

2 McCluskey, A. J., Olive, A. J., Starnbach, M. N. & Collier, R. J. Targeting HER2-positive cancer cells with receptor-redirected anthrax protective antigen. *Molecular Oncology* **7**, 440-451, doi:10.1016/j.molonc.2012.12.003 (2013).

3 Mironova, K. E. *et al.* Genetically Encoded Immunophotosensitizer 4D5scFv-miniSOG is a Highly Selective Agent for Targeted Photokilling of Tumor Cells In Vitro. *Theranostics* **3**, 831-840, doi:10.7150/thno.6715 (2013).

4 Zhou, J., Liu, Z. & Li, F. Upconversion nanophosphors for small-animal imaging. *Chemical Society Reviews* **41**, 1323-1349, doi:10.1039/c1cs15187h (2012).

5 Nadort, A. *et al.* Quantitative Imaging of Single Upconversion Nanoparticles in Biological Tissue. *Plos One* **8**, doi:10.1371/journal.pone.0063292 (2013).

6 Laqua, K., Melhuish, W. H. & Zander, M. NOMENCLATURE, SYMBOLS, UNITS AND THEIR USAGE IN SPECTROCHEMICAL ANALYSIS .7. MOLECULAR ABSORPTION-SPECTROSCOPY, ULTRAVIOLET

AND VISIBLE (UV VIS) - (RECOMMENDATIONS 1988). *Pure and Applied Chemistry* **60**, 1449-1460, doi:10.1351/pac198860091449 (1988).

7 Tikhomirov, V. K. *et al.* Cathodo- and photoluminescence in Yb3+-Er3+ co-doped PbF2 nanoparticles. *Optics Express* **18**, 8836-8846, doi:10.1364/oe.18.008836 (2010).

8 Boyer, J. C. & van Veggel, F. Absolute quantum yield measurements of colloidal NaYF4: Er3+, Yb3+ upconverting nanoparticles. *Nanoscale* **2**, 1417-1419, doi:10.1039/c0nr00253d (2010).

9 Zhao, J. B. *et al.* Single-nanocrystal sensitivity achieved by enhanced upconversion luminescence. *Nature Nanotechnology* **8**, 729-734, doi:10.1038/nnano.2013.171 (2013).

10 Vinegoni, C. *et al.* Transillumination fluorescence imaging in mice using biocompatible upconverting nanoparticles. *Optics Letters* **34**, 2566-2568 (2009).

11 Chen, G. *et al.* (alpha-NaYbF4:Tm3+)/CaF2 Core/Shell Nanoparticles with Efficient Near-Infrared to Near-Infrared Upconversion for High-Contrast Deep Tissue Bioimaging. *Acs Nano* **6**, 8280-8287, doi:10.1021/nn302972r (2012).

12 Grebenik, E. A. *et al.* Feasibility study of the optical imaging of a breast cancer lesion labeled with upconversion nanoparticle biocomplexes. *Journal of Biomedical Optics* **18**, doi:10.1117/1.jbo.18.7.076004 (2013).

13 Guller, A. E. *et al.* Cytotoxicity and non-specific cellular uptake of bare and surface-modified upconversion nanoparticles in human skin cells. *Nano Research* **8**, 1546-1562, doi:10.1007/s12274-014-0641-6 (2015).

14 Hwang, S. H. *et al.* Upconversion nanoparticle-based Forster resonance energy transfer for detecting the IS6110 sequence of Mycobacterium tuberculosis complex in sputum. *Biosens Bioelectron* **53**, 112-116, doi:10.1016/j.bios.2013.09.011 (2014).

15 Krammer, B. Vascular effects of photodynamic therapy. *Anticancer Research* **21**, 4271-4277 (2001).

16 Liu, D. L. *et al.* Tumour vessel damage resulting from laser-induced hyperthermia alone and in combination with photodynamic therapy. *Cancer Letters* **111**, 157-165, doi:10.1016/s0304-3835(96)04528-4 (1997).

17 Preise, D., Scherz, A. & Salomon, Y. Antitumor immunity promoted by vascular occluding therapy: lessons from vascular-targeted photodynamic therapy (VTP). *Photochemical & Photobiological Sciences* **10**, 681-688, doi:10.1039/c0pp00315h (2011).

18 Popov, A. P. *et al.* in *Novel Biophotonic Techniques and Applications Ii* Vol. 8801 *Proceedings of SPIE* (eds A. Vitkin & A. Amelink) (2013).
